# Allopregnanolone and perampanel as adjuncts to midazolam for treating diisopropylfluorophosphate‐induced status epilepticus in rats

**DOI:** 10.1111/nyas.14479

**Published:** 2020-09-11

**Authors:** Ashish Dhir, Donald A. Bruun, Michelle Guignet, Yi‐Hua Tsai, Eduardo González, Jonas Calsbeek, Joan Vu, Naomi Saito, Daniel J. Tancredi, Danielle J. Harvey, Pamela J. Lein, Michael A. Rogawski

**Affiliations:** ^1^ Department of Neurology, School of Medicine University of California, Davis Sacramento California; ^2^ Department of Molecular Biosciences, School of Veterinary Medicine University of California, Davis Davis California; ^3^ Department of Public Health Sciences, School of Medicine University of California, Davis Davis California; ^4^ Department of Pediatrics, School of Medicine University of California, Davis Sacramento California

**Keywords:** organophosphate nerve agent, status epilepticus, AMPA receptor antagonist, GABA_A_ receptor positive allosteric modulator, neuroactive steroid, benzodiazepine

## Abstract

Combinations of midazolam, allopregnanolone, and perampanel were assessed for antiseizure activity in a rat diisopropylfluorophosphate (DFP) status epilepticus model. Animals receiving DFP followed by atropine and pralidoxime exhibited continuous high‐amplitude rhythmical electroencephalography (EEG) spike activity and behavioral seizures for more than 5 hours. Treatments were administered intramuscularly 40 min after DFP. Seizures persisted following midazolam (1.8 mg/kg). The combination of midazolam with either allopregnanolone (6 mg/kg) or perampanel (2 mg/kg) terminated EEG and behavioral status epilepticus, but the onset of the perampanel effect was slow. The combination of midazolam, allopregnanolone, and perampanel caused rapid and complete suppression of EEG and behavioral seizures. In the absence of DFP, animals treated with the three‐drug combination were sedated but not anesthetized. Animals that received midazolam alone exhibited spontaneous recurrent EEG seizures, whereas those that received the three‐drug combination did not, demonstrating antiepileptogenic activity. All combination treatments reduced neurodegeneration as assessed with Fluoro‐Jade C staining to a greater extent than midazolam alone, and most reduced astrogliosis as assessed by GFAP immunoreactivity but had mixed effects on markers of microglial activation. We conclude that allopregnanolone, a positive modulator of the GABA_A_ receptor, and perampanel, an AMPA receptor antagonist, are potential adjuncts to midazolam in the treatment of benzodiazepine‐refractory organophosphate nerve agent–induced status epilepticus.

## Graphical abstract

In the present study, we demonstrate the potential of allopregnanolone, a positive modulator of the GABA_A_ receptor, and perampanel, an AMPA receptor antagonist, as adjuncts to the benzodiazepine midazolam in the treatment of organophosphate nerve agent–induced status epilepticus using the rat diisopropylfluorophosphate model.

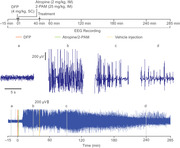

## Introduction

Acute intoxication with organophosphate (OP) nerve agents is associated with a life‐threatening peripheral cholinergic toxidrome^1^ and the acute development of seizures and refractory status epilepticus (SE).^2^ The seizures, which are believed to result from activation of muscarinic M1 receptors and excessive release of glutamate,^3^, ^4^ can lead to irreversible neuronal damage^5^, ^6^, ^7^ and epileptogenesis.^2^, ^8^, ^9^ The current standard‐of‐care treatment for OP poisoning—the muscarinic antagonist atropine sulfate and an oxime cholinesterase reactivator—only addresses the peripheral symptoms. Benzodiazepines are used to treat acute seizures. Midazolam (MDZ) recently received approval by the U.S. Food and Drug Administration (FDA) as a treatment for SE and is expected to be deployed as the antiseizure drug of choice in acute OP intoxication when an autoinjector formulation becomes available.^10^ Benzodiazepines may represent an effective countermeasure if administered within about 10 min after initiation of seizures, but are generally ineffective in terminating established SE (30 min or more after onset).^4^, ^11^, ^12^ Acute intoxication with diisopropylfluorophosphate (DFP), an OP considered to be a credible threat agent,^13^ causes SE that is not terminated by the administration of MDZ 40 min or later after intoxication.^14^ Additionally, delayed treatment with MDZ does not prevent epileptogenesis caused by OP‐induced SE.^12^

Refractoriness to the antiseizure efficacy of benzodiazepines during prolonged seizures may be caused by internalization of synaptic GABA_A_ receptors.^15^, ^16^ Extrasynaptic GABA_A_ receptors, which are insensitive to benzodiazepines, are not internalized with prolonged seizures, and they provide a therapeutic target for refractory SE, including that caused by OP nerve agents.^17^ Allopregnanolone (5α‐pregnan‐3α‐ol‐20‐one; brexanolone; ALLO), an endogenous neuroactive steroid that acts as an allosteric modulator of both synaptic and extrasynaptic GABA_A_ receptors, has efficacy in diverse seizure models,^18^ including models of SE.^19^, ^20^, ^21^ The closely related synthetic neuroactive steroid ganaxolone has been shown to be effective in managing seizures induced by DFP and other OPs.^21^, ^22^ There is also evidence that ALLO may have antiepileptogenic properties.^23^, ^24^

Excessive glutamate excitation is a significant mediator of OP SE, and the neuropathology associated with OP poisoning is largely a consequence of glutamatergic excitotoxicity.^25^ Diverse glutamate receptor antagonists have been proposed as treatments for OP nerve agent seizures, including the mixed AMPA and GluK1 kainate receptor antagonist tezampanel (LY293558)^26^ and the NMDA receptor antagonists ketamine and caramiphen.^27^, ^28^, ^29^ While GluK1 kainate receptors have not been confirmed as pertinent antiseizure targets,^30^ NMDA receptor antagonists may be effective clinically^31^ and have been shown in animal models to be effective in treating OP seizures.^32^, ^33^ However, NMDA receptors contribute only partially to fast glutamate‐mediated synaptic transmission, and NMDA receptor antagonists, such as ketamine, are associated with adverse neurobehavioral effects.^34^ AMPA receptors contribute more than NMDA receptors to synaptic excitation and pharmacological blockade of AMPA receptors is not associated with ketamine‐like adverse effects. It is, therefore, of interest to evaluate the inclusion of an AMPA receptor antagonist in the treatment regimen for OP‐induced SE. Perampanel (PPL) is a potent, highly selective noncompetitive AMPA receptor antagonist approved for epilepsy therapy.^35^ Studies in animal models^36^ and anecdotal evidence from numerous case reports indicate utility in the treatment of SE.^37^, ^38^, ^39^ Moreover, AMPA receptor antagonists, including PPL, can depress epileptogenesis,^40^ including that caused by prolonged SE.^41^

A consistent finding in studies of the neurotoxicological effects of OP nerve agents is that acute OP‐induced seizures cause progressive neurodegeneration and neuroinflammation in the days to weeks following exposure.^7^, ^42^, ^43^, ^44^, ^45^ Treatment with benzodiazepines, even when administered early in the course of SE, does not protect completely against ensuing brain injury.^45^, ^46^, ^47^, ^48^ The severity and extent of brain damage correlates directly with the intensity, frequency, and duration of seizures^42^, ^43^, ^49^ Thus, it is widely posited that long‐term outcomes will be significantly improved by antiseizure therapies that more effectively terminate SE and prevent seizure recurrence.^50^, ^51^, ^52^

In the present study, we assess the potential of ALLO and PPL as adjuncts to MDZ in the treatment of OP‐induced SE using the rat DFP model. Our results demonstrate that both agents enhance the activity of MDZ. In a previous study, we showed that epileptogenesis occurs in rats that survive DFP seizures,^7^ and we now provide evidence that adjunctive treatment with the combination of ALLO and PPL has antiepileptogenic potential.

## Materials and methods

### Animals

All experiments involving animals complied with the ARRIVE guidelines of the National Centre for the Replacement, Refinement and Reduction of Animals in Research and were performed in accordance with the National Institutes of Health Guide for the Care and Use of Laboratory Animals (NIH publication No. 8023, revised in 1978) under protocols approved by the University of California, Davis, Institutional Animal Care and Use Committee.

Adult male Sprague–Dawley rats (225–400 g) were purchased at 8–10 weeks of age from Charles River Laboratories (Hollister, CA) and individually housed in standard plastic cages under controlled environmental conditions (22 ± 2 °C, 40–50% humidity) in fully accredited AALAC International facilities. Animals were housed in a 12 h light‐dark cycle with access to food and water provided *ad libitum*. Electroencephalography (EEG) experiments were performed during the light phase of the light/dark cycle after a minimum 30 min period of acclimation to the experimental room. In experiments involving measurement of spontaneous recurrent seizures (SRS), animals were additionally measured for their electrographic seizures in the dark cycle.

### Test substance and drug administration

DFP, atropine sulfate, and pyridine‐2‐aldoxime methochloride (pralidoxime chloride; 2‐PAM) were purchased from Sigma‐Aldrich (St. Louis, MO). A commercially available formulation of midazolam (MDZ; 5 mg/mL) was obtained from Hospira Inc. (Lake Forest, IL). ALLO was synthesized under contract with SAFC Pharma (Madison, WI). PPL was a generous gift from Eisai Inc. (Fycompa^®^; E2007; Woodcliff Lake, NJ).

Before use, DFP was confirmed to be 90 ± 7% pure, as determined using previously published methods.^53^ DFP was aliquoted and stored at −80 °C; under these conditions, it has been shown that DFP is stable for at least 400 days.^54^ Five minutes before administration, DFP was diluted in sterile, ice‐cold phosphate‐buffered saline (PBS; 3.6 mM Na_2_HPO_4_, 1.4 mM NaH_2_PO_4_, 150 mM NaCl, pH 7.2) and administered subcutaneously at a dose of 4 mg/kg in a volume of 300 microliters. Atropine sulfate (2 mg/kg) and 2‐PAM (25 mg/kg) in sterile saline were administered in a combined intramuscular injection 1 min following injection of DFP to prevent lethality from peripheral cholinergic symptoms.^55^ MDZ was administered as the commercial 5 mg/mL solution for injection (Hospira). ALLO (6 mg/mL) was dissolved in 24% w/v Captisol^®^ (10% sulfobutyl ethers β‐cyclodextrin sodium salts) in 0.9% w/v NaCl. A 4 mg/mL solution of PPL was prepared using a multisol consisting of 7:2:1 propylene glycol:ethanol:water. ALLO and PPL were injected in dose volumes of 1 and 0.5 mL/kg, respectively. The treatments were administered intramuscularly, and in the case of combinations, the injections were delivered in rapid succession (separated by ∼5 seconds). In vehicle control groups, three separate injections were used to administer the vehicles for MDZ, ALLO, and PPL, which were, respectively, 0.9% w/v NaCl; 24% w/v Captisol^®^ in 0.9% w/v NaCl; and the 7:2:1 propylene glycol, ethanol, and water mixture.

### EEG electrode implantation

Animals were anesthetized using ketamine (60–80 mg/kg IP) and dexmedetomidine (0.5–1 mg/kg IP) and stabilized in a stereotaxic apparatus. Six ⅛ inch bone screws with wire leads (#8247, Pinnacle Technology, Lawrence, KS) were implanted epidurally, three on each side of the sagittal skull suture. A 6‐pin rat prefabricated head‐mount implant (mounted on a 9 mm × 9 mm board with EEG electrode wires attached; #8239‐SE3, Pinnacle Technology) was soldered to the screws and fixed to the skull using dental acrylic cement. The skin was sutured, and the animals were administered ketoprofen (5 mg/kg, SC; Ketofen^®^, Zoetis, Parsippany, NJ) during the surgery and the following day. Anesthesia was reversed by atipamezole (1 mg/kg, SC; Antisedan^®^, Zoetis). Animals were returned to the vivarium and allowed a minimum of 7–10 days for postsurgery recovery, during which time they received comprehensive veterinary care. Following recovery, animals were brought into the laboratory and acclimatized for at least 30 min before the actual experiment.

**Figure 1:**
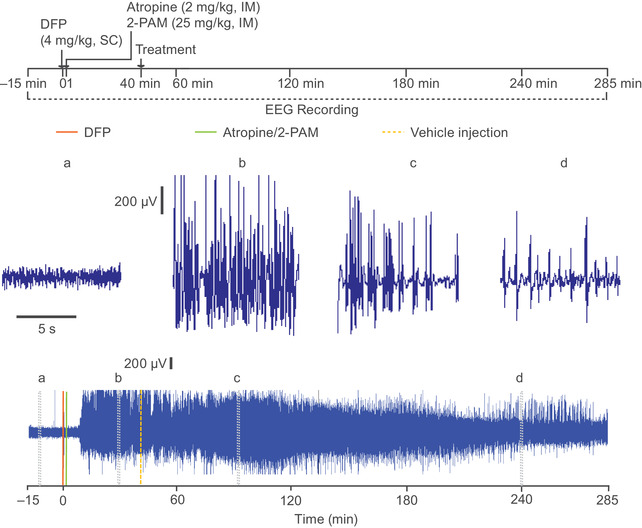
Schematic illustration of the experimental protocol for the DFP status epilepticus (SE) model (top panel) and EEG recording showing full‐blown SE with vehicle treatment at 40 min (bottom panel). DFP was injected at time 0 followed 1 min later by injections of atropine and 2‐pralidoxime chloride (2‐PAM). Forty minutes after DFP challenge, animals received three vehicle injections as described in the Methods. The entire EEG record is shown in the lower trace; short segments of the record on an expanded time scale from the regions marked with letters (a–d) are shown above. High‐amplitude rhythmical spiking occurs continuously after DFP treatment.

**Figure 2:**
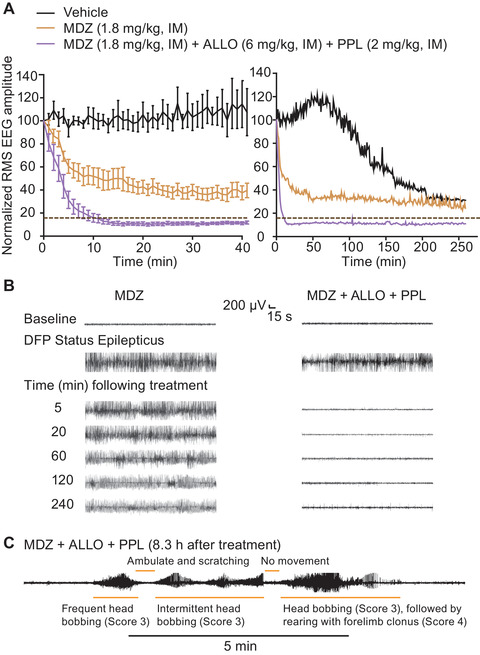
Comparison of vehicle, MDZ, or the combination of MDZ, ALLO, and PPL on DFP‐induced electrographic SE. (A) Mean normalized (percentage) RMS amplitude values for the first 40 min after treatment are shown in the left graph and for the entire recording period in the right graph. Each data point represents the mean of values from 8 to 10 rats. Error bars in the left graph represent S.E.M values; they are omitted for clarity on the right. Baseline normalized RMS amplitude (15.9%) is indicated by dashed lines. (B) Representative EEG segments at various times after treatment in an experiment with MDZ (left) or the combination (right). High‐amplitude rhythmic spiking was not terminated by MDZ, whereas the combined therapy terminated SE in all animals. After SE termination, some animals in the latter group exhibited aperiodic high‐amplitude spike‐like phenomena, possibly representing burst suppression activity. (C) Representative EEG segment demonstrating SE relapse in one of the four animals in the MDZ + ALLO + PPL group that underwent long‐term monitoring. Orange bars indicate periods of apparent seizure and nonseizure behaviors. [Correction added on September 18, 2020, after online publication: Figure 2 was replaced with the correct version.]

**Figure 3:**
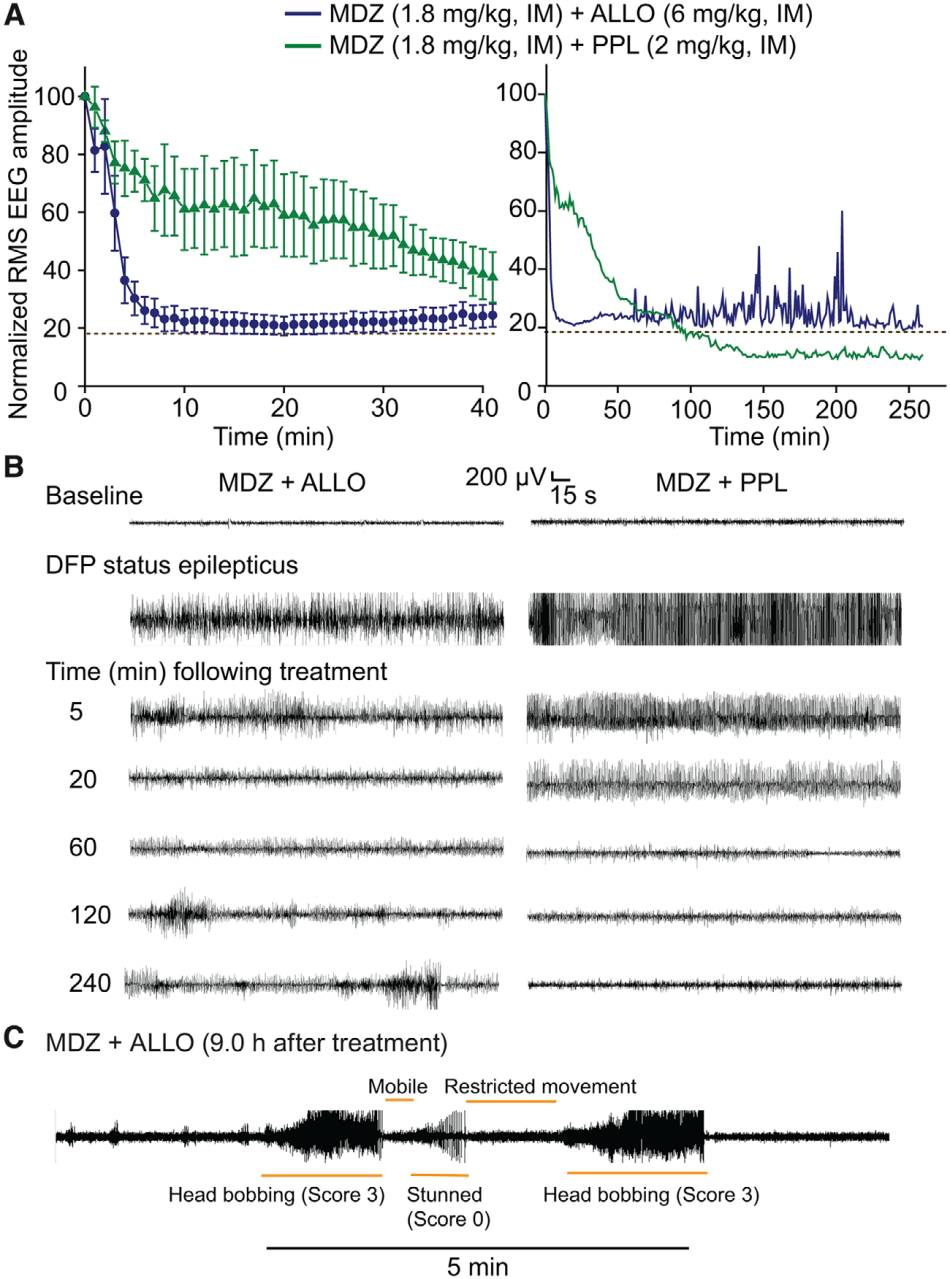
Effect of MDZ combined with either ALLO or PPL on DFP‐induced electrographic SE. (A) Mean normalized (percentage) RMS amplitude values for the first 40 min after the treatment are shown in the left graph and for the entire recording period in the right graph. Each data point represents the mean of values from 6 to 10 rats. Error bars in the left graph represent S.E.M. values; they are omitted for clarity in the right. Baseline normalized RMS amplitude (18.8%) is indicated by the dashed line. (B) Representative EEG segments at various times after treatment with MDZ + ALLO (left) and MDZ + PPL (right). Treatment with MDZ + ALLO rapidly stopped electrographic seizures in eight of 10 animals; however, in two animals, SE persisted as shown here. In some of those animals in which SE stopped, aperiodic high‐amplitude spike‐like phenomenon possibly representing burst suppression activity was observed. The combination of MDZ + PPL provided delayed protection in stopping SE. (C) Representative EEG segment demonstrating SE relapse in one out of five animals in the MDZ + ALLO group that underwent long‐term monitoring. Orange bars indicate periods of apparent seizure and nonseizure behaviors.

Signals were differentially recorded between the following EEG electrode pairs: (1) left parietal and left prefrontal, (2) right parietal and left prefrontal, and (3) right posterior hippocampus and left posterior hippocampus. During experiments, signals from all three pairs were continuously monitored to provide an ongoing assessment of seizure activity. The signals displayed in Figures 1, 2, 3 and subjected to analysis were from the electrode pair (1).

### DFP‐induced SE model and drug treatments

Rats with implanted electrodes were randomly divided into treatment groups and individually housed in cages in which they could move freely. At the time of the experiment, they were weighed and tethered to a Pinnacle EEG monitoring system. Animals were injected with DFP followed by atropine and 2‐PAM as described above; 40 min after the DFP injection, animals confirmed to be in SE using behavioral criteria and by EEG received one of the following treatments by intramuscular injection: (1) vehicle solutions, *n* = 8; (2) MDZ (1.8 mg/kg), *n* = 10; (3) MDZ (1.8 mg/kg) + ALLO (6 mg/kg), *n* = 10; (4) MDZ (1.8 mg/kg) + PPL (2 mg/kg), *n* = 6; (5) MDZ (1.8 mg/kg) + ALLO (6 mg/kg) + PPL (2 mg/kg), *n* = 11; and (6) ALLO (6 mg/kg) + PPL (2 mg/kg), *n* = 5.

### Behavioral seizure assessment

**Figure 4:**
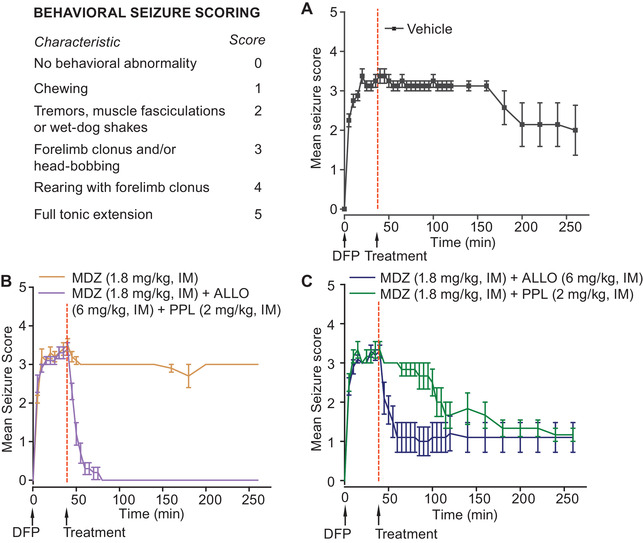
Effects of different treatments on behavioral seizure scores in the DFP SE model. The seizure scoring scheme based on a modification of the Racine Scale is shown in the upper left panel. (A) After DFP exposure (and with vehicle treatment at 40 min), the mean behavioral seizure score rises rapidly and remains elevated for about 150 min and then begins to decline. (B) MDZ has little effect on the mean seizure score, whereas MDZ + ALLO + PPL treatment caused a rapid reduction in seizure score in all animals by 10 min following treatment. (C) MDZ + ALLO and MDZ + PPL reduced the mean seizure scores more than MDZ alone. In the MDZ + ALLO group, the score was reduced to 0 in eight of 10 animals, while the remaining two animals continued to exhibit behavioral seizures. The onset of action of MDZ + PPL was delayed compared with MDZ + ALLO. Data points represent the mean ± S.E.M. values from experiments with 6–10 rats.

Behavioral seizures were scored based on a modified Racine Score as described by Deshpande *et al*.^56^ (see Fig. 4 inset). Score 1 (chewing) and score 2 (tremors, muscle fasciculation, and wet‐dog shakes) behaviors were generally not associated with electrographic seizures and may reflect the peripheral cholinergic effects of DFP. Scoring was conducted every 5 min for the first 120 min following a DFP challenge and then every 20 min for a total of 260 minutes. A 5% w/v dextrose solution was administered intraperitoneally 5 h after DFP treatment to counter fluid lost during continuous seizure activity. Animals were returned to their home cages and given soft chow daily until they were capable of consuming both food and water by themselves. Some animals were administered a second dose of the dextrose solution 24 h after DFP treatment. Animals were maintained in the vivarium and the number of animals that survived at the 7‐day time point was noted.

### Loss of righting reflex

Loss of righting reflex (LRR) was determined as described previously.^57^ Following intramuscular injection of MDZ + ALLO + PPL, rats were returned to their home cages. If an animal exhibited arrest of their spontaneous activity, they were turned on their back every 10 seconds. Failure to spontaneously return to an upright posture was scored as LRR. The difference (in seconds) between the time of LRR and the time an animal regained the ability to right themselves was reported as the total sleep time.

### EEG measurement and analysis

EEG signals were recorded with a Pinnacle Technology 3‐EEG rat preamplifier (#8407) and Data Conditioning and Acquisition System (#8401). The sampling frequency was 250 Hz, except in some initial experiments where 1000 Hz was used. High‐ and low‐pass filtering was set at 1 and 40 Hertz. We have found through power spectral analysis in the frequency domain that EEG power is increased during DFP seizures within frequency bands encompassed within the 1‐ to 40‐Hz range of frequencies. A similar range of frequencies has been used to assess drug effects on DFP seizures by others.^4^, ^58^, ^59^ It was desirable to eliminate higher frequencies, which may represent electrical noise and other artifacts, including muscle movement.^60^ Rats were allowed to move freely in the monitoring system during recordings. After baseline recordings were obtained for at least 15 min, animals were injected with DFP and EEG recording continued for at least 5 hours. Some animals in each group (except MDZ + PPL) were monitored continuously for 2–3 days for seizure progression. Recordings were reviewed using Sirenia Seizure Pro software (Pinnacle Technology). The root mean square (RMS; square root of the mean value of the squared values of the voltage amplitude) was calculated for each 1 min‐epoch of the EEG using SIGVIEW spectrum analyzer software, version 3.0.2 (SignalLab e.K., Pforzheim, Germany). The RMS value is a useful discriminator of seizure versus nonseizure activity.^61^, ^62^ To account for arbitrary differences in RMS amplitude caused, for example, by variations in electrode position or signal detection efficiency, the RMS amplitude values were normalized to the RMS amplitude value at the initiation of treatment, which is 40 min after administration of DFP, and expressed as a percentage. In addition to the RMS amplitude analysis, we visually inspected the EEG traces to identify the initiation and termination of SE and the occurrence of isolated seizures. Consistent with the methodology used by others,^8^, ^63^ ictal activity was scored when high‐frequency rhythmic spike‐wave discharges occurred with RMS amplitude ≥two‐fold the baseline signal for at least 5 s, as previously defined. Electrographic SE was defined as ictal activity for 5 min or more and termination of electrographic SE was defined as cessation of EEG seizure activity for more than 5 minutes.

### Spontaneous recurrent seizure recording and measurement

Following DFP exposure, animals were monitored for SRS using the Pinnacle tethered EEG system. Animals that had been returned to the vivarium after the acute study were brought back to the laboratory on random days up to 7.5 months after DFP exposure for EEG recording to determine the occurrence of SRS. A seizure in a freely moving animal was defined as a spike‐wave discharge at a frequency >5 Hz for at least 5 s, where the amplitude of the spikes are ≥two‐fold the background EEG level. EEG recordings were conducted in sessions mainly during the morning but in some cases, the session extended later in the day. The records were scored manually for seizures and the following values were determined in each recording: (1) seizure rate calculated by dividing the seizure count by the recording time (in hours), and (2) seizure burden calculated as the percentage of the recording time in seizure activity. The seizure rate and seizure burden were determined for two time periods: (1) the initial 4 days after DFP injection and (2) the remainder of observation period. A limitation of our approach is that we did not continuously monitor animals and therefore may have missed spontaneous seizures in some animals. We attempted to mitigate this concern in this study by comparing groups treated in the same manner.

### Immunohistochemistry

For histology, tissues were collected at 3, 7, and 28 days after DFP exposure (sample sizes indicated in figure legends). Previous studies using the rat model of acute DFP intoxication have demonstrated significant neurodegenerative and neuroinflammatory responses at these time points^44^, ^64^ and showed that these effects were modestly attenuated by treatment with benzodiazepines administered 30 min or more postexposure.^14^, ^60^, ^61^ Rats were deeply anesthetized with 4% isoflurane in medical grade O_2_ at a flow rate of 1 L/min followed by transcardial perfusion with 100 mL cold PBS using a Masterflex peristaltic pump (Cole‐Parmer, Vernon Hills, IL) at a flow rate of 15 mL/min until the perfusate ran clear. Brains were removed from the skull, blocked into 2‐mm‐thick coronal sections using a brain matrix and postfixed for 24 h at 4 °C in 4% paraformaldehyde (PFA; Sigma‐Aldrich) in phosphate buffer (0.1 M Na_2_HPO_4_, 0.1 M NaH_2_PO_4_, pH 7.2). Tissues were then transferred to 30% (w/v) sucrose (Sigma‐Aldrich) in PBS and stored at 4 °C for at least 48 h until embedded and flash frozen in Tissue‐Plus™ O.C.T. compound (Thermo Fisher Scientific, Waltham, MA). Tissue blocks were cryosectioned into 10‐μm‐thick coronal sections and stored at −80 °C until immunostained.

All slides were processed at the same time to reduce staining variability among treatment groups. For Fluoro‐Jade C (FJC; Chemicon, Temecula, CA) staining, slides were stained following the manufacturer's recommended protocol as previously described.^64^ For other biomarkers, slides were brought to room temperature, washed with PBS, placed in 10 mM sodium citrate buffer at pH 6.0, and heated for 30 min in a rice cooker for antigen retrieval. Following antigen retrieval, sections were incubated in blocking buffer, which was 10% (v/v) normal goat serum (Vector Laboratories, Burlingame, CA), 1% (w/v) bovine serum albumin (Sigma‐Aldrich), and 0.3% (v/v) Triton X‐100 (Thermo Fisher Scientific) in PBS, for 1 h at room temperature, and then incubated with primary antibody in blocking buffer at 4 °C overnight. Primary antibodies included rabbit anti‐IBA1 (1:1000, 019–19741, Wako Laboratory Chemicals, Richmond, VA), mouse anti‐CD68 (1:200, MCA341R, Serotec, Hercules, CA), mouse anti‐GFAP (1:1000, 3670, Cell Signaling Technology, Danvers, MA), and mouse anti‐NeuN (1:1000, MAB377, Millipore, Burlington, MA). Slides were washed three times for 10 min each in PBS with 0.3% (v/v) Triton X‐100 and then incubated in the secondary antibody in blocking buffer for 2 h at room temperature. The secondary antibody used to detect anti‐IBA1 was goat anti‐rabbit IgG conjugated to Alexa Fluor^®^ 568 nm (1:500, 997761, Life Technologies, Carlsbad, CA); to detect anti‐CD68, goat anti‐mouse IgG conjugated to Alexa Fluor 488 nm (1:500, A11001, Life Technologies); and to detect anti‐GFAP or anti‐NeuN, goat anti‐mouse IgG1(γ1) conjugated to Alexa Fluor 568 nm (1:1000, A21124, Life Technologies). All slides were mounted in Prolong Gold anti‐fade mounting media with DAPI (Invitrogen).

Fluorescent images were acquired using the ImageXpress Micro XLS Widefield High‐Content Analysis System (Molecular Devices, Sunnyvale, CA). Positive immunostaining was identified as fluorescence intensity that was twice the background fluorescence levels in negative control samples. Images were acquired from the basolateral amygdala (bregma −3.6 to −4.2), dorsolateral thalamus (bregma −3.0 to −3.6), piriform cortex (bregma −3.6 to −4.2), hippocampus (bregma −3.6 to −4.2), and somatosensory cortex (bregma −3.6 to −4.2) at the same coronal plane across all animals as determined using a photographic rat brain atlas.^65^ Two serial slices were analyzed for each brain region of each animal. Multiple overlapping tiles were stitched together to produce a single image that encompassed an entire brain region that was then processed using automated analysis platforms to minimize operator bias. A MATLAB script (MATLAB^®^ 2014b, the Mathworks Inc., Natick, MA) was used to set masking levels for each image, so that only positive stained cells were visualized, and then fluorescence was measured per area using software from the Custom Module Editor analysis software (MetaXpress High‐Content Image Acquisition and Analysis software, version 5.3, Molecular Devices).

### Statistics

EEG traces in each animal were summarized in 1‐min epochs as the mean RMS amplitude value of (250 Hz sampling frequency × 60 s) = 15,000 values/minute. At minute 40, animals were administered treatments. The mean RMS amplitude values at later time points were normalized to the minute 40 value, expressed as a percentage. Two vehicle control animals did not survive during the 5‐h observation period; one perished at minute 168 and the other at minute 246. The missing minutes for these animals were filled out with their last observed value (last observation carried forward), a conservative strategy because the last observed value for each animal was among that animal's lowest.

To statistically compare treatment groups based on the EEG data, between‐group comparisons of mean within‐animal changes were performed for each of three posttreatment time periods (hours 1, 2, and 3^+^) relative to the 40‐min pretreatment time period. Mixed‐effects models were applied to a dataset where the units of analysis were within‐animal time point–specific averages (i.e., four observations per animal). Models were estimated with Version 9.4 of SAS, using the PROC GLIMMIX procedure, specifying a log‐normal model with random intercepts for each animal (to adjust for between‐animal differences) and autocorrelated residual errors, which adjust for the feature in longitudinally measured data that transient measurement errors from observations from the same animal that are closer in time will be more correlated than from observations from the same animal that are further apart in time. Because groups were compared with respect to mean within‐group changes, statistical adjustment was possible for between‐group differences prior to treatment. Additionally, how each posttreatment time point compares with the pretreatment time point is expressed as a geometric mean ratio.

In the analysis of epileptogenesis, data for seizure rate and seizure burden were visualized using dot plots (shown) and panel data plots (not shown). Because parametric modeling assumptions for *t*‐test and ANOVA were not met, between‐group comparisons at each time point were carried out by plotting the observed data and using the nonparametric Mann–Whitney test for the null hypothesis that the observations come from the same observation.

For analysis of the immunohistochemical data, repeated measures models, including treatment (MDZ alone, MDZ + ALLO, MDZ + PPL, MDZ + ALLO + PPL, or ALLO + PPL), region (cortex, CA1, CA3, dentate gyrus, thalamus, amygdala, or piriform cortex), and time point (3, 7, and 28 days postexposure), were fit to the immunohistochemical endpoints (percent area for GFAP, FJC, IBA‐1, and CD68 and number of positive cells per mm^2^ for NeuN). Only two time points were available (3 and 28 days postexposure) for IBA‐1 and CD68. Interactions between the factors were considered, and the best model was chosen using the Akaike Information Criterion. All immunohistochemical endpoints were transformed using the natural logarithm to better meet the assumptions of the models; owing to observed zeros, all values for these endpoints were first shifted by 0.5 before taking the natural log. Results are presented as geometric mean ratios of the values for the combination treatments to the values for treatment with MDZ alone. These ratios may be interpreted as fold changes, so that a ratio of 1.5 corresponds to a 50% increase and a ratio of 0.5 corresponds to a 50% decrease. Point estimates of the ratios and 95% confidence intervals are presented in the figures. When the confidence interval includes 1, there is no statistical evidence of a difference between the combined treatment and treatment with MDZ alone.

## Results

### DFP‐induced behavioral and electrographic seizures in vehicle‐treated animals

Animals treated according to the DFP SE model (scheme shown in Fig. 1) exhibited robust electrographic seizures, as evidenced by the abrupt (typically within 6–9 min) onset of high‐amplitude rhythmical spiking (Fig. 1). If not pharmacologically interrupted, the epileptiform discharges continued for more than 5 h unless the animal expired during the observation period, which was the case for two out of eight (25%) animals in the vehicle control group (168 and 246 min after DFP injection). All of the vehicle‐treated animals that survived the initial observation period also survived for 7 days. Animals exhibiting DFP‐induced electrographic seizures displayed behavioral seizures consisting of continuous jerking movement, forelimb clonus, and rearing and falling (scored as 3 or 4 on the modified Racine Scale), and rarely tonic extension (scored as 5) (Fig. 4). Behaviors scored as 1 (chewing movements) and 2 (tremors, muscle fasciculations, and wet‐dog shakes) often occurred in the absence of demonstrable EEG seizure discharges, suggesting that they may be due to peripheral cholinergic effects of intoxication with DFP. We did not consider scores of 1 or 2 to be a reliable indicator of seizures. In vehicle‐treated animals, the mean behavioral seizure score remained high throughout most of the observation period but began to decline at 150 min (Fig. 4A), whereas the normalized RMS amplitude rose gradually during the first hour and then declined during the 250‐min observation period but remained above the baseline value (Fig. 2A). Most animals exhibited continuous high‐amplitude rhythmic spiking throughout the 5‐h recording period (Fig. 2B). However, EEG spikes continued even when the SE stopped in two animals, and most of the animals continued to experience seizures 24 h after DFP exposure (data not shown). The mean RMS values during the first and second hours after vehicle treatment were not significantly different from the 40 min period after DFP treatment and before vehicle injection (geometric mean ratios were 1.25 (95% confidence interval (CI): 0.95–1.64) and 1.08 (95% CI: 0.70–1.68), respectively). However, in the third hour, the mean RMS amplitude value was reduced compared with the 40‐min pretreatment time period (geometric mean ratio = 0.49, 95% CI: 0.25–0.96). We monitored two animals in this group for 3 days to assess the long‐term impact of DFP. In one of the animals, SE persisted for 2 days and then stopped. In the second animal, SE continued for 17 h, after which isolated spikes and seizures were observed, but there was no relapse of SE.

### MDZ is partially effective in suppressing DFP‐induced EEG and behavioral seizures

Some animals were treated with MDZ alone at a dose (1.8 mg/kg) that is equivalent by allometric scaling (according to the FDA guidance) to a 20 mg dose (maximum recommended adult dose; see Ref. 66) in a 70 kg person. The mean normalized RMS values of the MDZ treatment group diverged significantly from the vehicle control group but never reached the baseline value of freely moving control animals (Fig. 2A). MDZ treatment resulted in 2 out of 10 (20%) animals achieving SE termination at 114 and 262 min following the treatment. The remaining eight animals continued to experience seizures and SE throughout the recording period (Fig. 2B). MDZ caused a statistically significant reduction in the mean EEG RMS measurement during the 1st hour (geometric mean ratio = 0.54, 95% CI: 0.42–0.69, *P* < 0.0001), 2nd hour (geometric mean ratio = 0.40, 95% CI: 0.30–0.54, *P* < 0.0001), and the 3+ hour (geometric mean ratio = 0.37, 95% CI: 0.28–0.48, *P* < 0.0001) compared with the 40‐min pretreatment time period. Furthermore, the ratio of MDZ (*n* = 10) and vehicle (*n* = 8) geometric mean ratios were found to be statistically significantly <1 for the first two posttreatment hours (1st hour: effect size = 0.43, 95% CI: 0.30–0.62, *P* < 0.0001; 2nd hour: effect size = 0.37, 95% CI: 0.22–0.63, *P* = 0.0003), but not the 3+ hour (effect size = 0.75, 95% CI: 0.36–1.53, *P* = 0.4191), indicating that MDZ treatment suppresses the RMS EEG amplitude during each of the first 2 hourly epochs.

As depicted in Figure 4A and B, the behavioral seizure score of the MDZ‐treated animals was similar to the vehicle group and remained high throughout the observation period. Most animals continued to exhibit electrographic SE despite MDZ treatment. In one animal, however, SE in the EEG appeared to break at 114 minutes. This animal continued to exhibit frequent EEG spikes and was clearly in a continuing state of SE with a behavioral seizure score of 3.

We monitored four of the 10 MDZ‐treated animals for 3 days. In one of the animals, SE persisted for 5 h, while the other three continued to exhibit SE for 10–19 hours. Spikes and sporadic seizures were observed following SE termination in these animals. In one of the four animals, there was a relapse to SE 6 h after its initial termination that persisted for 1 hour.

### Addition of ALLO augments the antiseizure activity of MDZ

As seen in Figure 3A, treatment with MDZ + ALLO caused a rapid fall in the mean normalized RMS value to near to baseline. In eight of 10 animals (80%) receiving the drug combination, electrographic SE terminated with mean latency of 9.1 ± 2.1 min after the treatment, and behavioral seizures were also rapidly terminated (score 0) in most (eight of 10) animals (Fig. 4C). In the remaining two animals that continued to exhibit EEG SE, behavioral seizures persisted at a score of 3 and sometimes 4. In the entire group, there was a statistically significant reduction in the mean EEG RMS amplitude during the 1st hour (geometric mean ratio = 0.28, 95% CI: 0.21–0.38, *P* < 0.0001), 2nd hour (geometric mean ratio = 0.24, 95% CI: 0.17–0.33, *P* < 0.0001), and 3+ h (geometric mean ratio = 0.25, 95% CI: 0.16–0.39, *P* < 0.0001) compared with the 40‐min pretreatment time period. None of the animals receiving the treatment died during the 7‐day observation period. One animal developed a large abdominal tumor at day 162 and was euthanized. Figure 3B (left) depicts a representative EEG from one of the two animals in which SE did not stop with MDZ + ALLO treatment. The animal continued to exhibit high‐amplitude rhythmic spiking throughout the recording period. The ratio of the MDZ + ALLO (*n* = 10) and MDZ (*n* = 10) geometric mean ratios are statistically significantly <1 for the first two posttreatment epochs (1st hour: effect size = 0.53, 95% CI: 0.36–0.77, *P* = 0.0010; 2nd hour: effect size = 0.59, 95% CI: 0.38–0.93, *P* = 0.0216), but not the third (3+ hour: effect size = 0.69, 95% CI: 0.41–1.15, *P* = 0.1542), indicating that MDZ+ALLO treatment suppresses the RMS EEG amplitude during each of the first 2 hourly episodes to a greater extent than MDZ alone. Comparing the vehicle and MDZ + ALLO groups, the geometric mean ratios are statistically significant for the first and second but not the third epochs (1st hour: effect size = 0.23, 95% CI: 0.15–0.34, *P* < 0.0001; 2nd hour: effect size = 0.22, 95% CI: 0.13–0.38, *P* < 0.0001; 3+ hour: effect size = 0.51, 95% CI: 0.23–1.14, *P* = 0.0999). We monitored 5 of the 10 MDZ + ALLO animals for 3 days. SE in four of the animals stopped within minutes of treatment but one animal exhibited continuing SE for 13 hours. Following the termination of SE, three of the five monitored animals had isolated seizures, and one animal had relapses of SE beginning at 9 h (Fig. 3C). In that animal, such relapses continued into the following day.

### Addition of PPL to MDZ has mixed effects

In contrast with the results with ALLO, addition of PPL to MDZ did not rapidly suppress either EEG or behavioral seizures, although seizures were slowly suppressed relative to vehicle. Comparing Figures 3A and 2A, it is apparent that the mean normalized RMS values in the PPL + MDZ group actually exceeded those in the MDZ group in the first 40 min after treatment, indicating a paradoxical early proconvulsant action relative to MDZ, which is evident in the EEG traces in Figure 3B (right panel). However, at 40 min, the curve for the MDZ + PPL group falls below vehicle, and then at 100 min falls below the baseline value. The mean SE stopping time was 93.0 ± 14.5 (individual animal stopping times: 49, 62, 88, 91, 133, and 135 minutes). In the MDZ + PPL group, there was a statistically significant reduction in the mean EEG RMS amplitude during the 1st hour (geometric mean ratio = 0.54, 95% CI: 0.38–0.76, *P* = 0.0006), 2nd hour (geometric mean ratio = 0.20, 95% CI: 0.12–0.34, *P* < 0.0001), and the 3+ hour (geometric mean ratio = 0.12, 95% CI: 0.11–0.13, *P* < 0.0001) compared with the 40‐min pretreatment time period. The ratio of the MDZ + PPL (*n* = 6) and MDZ (*n* = 10) geometric mean ratios are statistically significantly <1 for the last two epochs (2nd hour: effect size = 0.49, 95% CI: 0.27–0.90, *P* = 0.0218; 3+ hour: effect size = 0.33, 95% CI: 0.25–0.44, *P* < 0.0001), but not the first epoch, which included the initial 40‐min period of greater RMS amplitude (1st hour: effect size = 1.00, 95% CI: 0.66–1.53, *P* = 0.9936).

As shown in Figure 4C, the behavioral seizures only fell minimally during the 40‐min post‐treatment period and then fell more rapidly at 100 min but then plateaued such that on average, seizures were never eliminated. This contrasts with the situation for MDZ + ALLO, where many animals achieved seizure freedom (seizure score 0) but because two did not, the mean score value remained elevated. In the MDZ + PPL group, there was no mortality on the day of recording, but two of six animals died within 7 days of the experiment.

### Simultaneous administration of MDZ, ALLO, and PPL rapidly eliminates EEG and behavioral seizures

As shown in Figure 2A, the combination MDZ + ALLO + PPL caused a rapid fall in mean normalized RMS amplitude similar to that seen with MDZ + ALLO and at 10 min following treatment became minimally smaller than baseline, where it remained during the observation period. There was a statistically significant reduction in the mean EEG RMS amplitude during the 1st hour (geometric mean ratio = 0.22, 95% CI: 0.18–0.26, *P* < 0.0001), 2nd hour (geometric mean ratio = 0.14, 95% CI: 0.12–0.17, *P* < 0.0001), and the 3+ hour (geometric mean ratio = 0.14, 95% CI: 0.12–0.16, *P* < 0.0001) compared with the 40‐min pretreatment time period. The ratio of ALLO + PPL + MDZ (*n* = 9) and MDZ (*n* = 10) geometric mean ratios are statistically significantly <1 for all the three epochs (1st hour: effect size = 0.40, 95% CI: 0.29–0.55, *P* < 0.0001; 2nd hour: effect size = 0.35, 95% CI: 0.25–0.49, *P* < 0.0001; 3rd hour: effect size = 0.37, 95% CI: 0.27–0.50, *P* < 0.0001). The mean latency to cessation of EEG SE in the ALLO + PPL + MDZ group was 13.0 ± 2.6 minutes. A representative EEG is shown in Figure 2B. As shown in Figure 4B, ALLO + PPL + MDZ also rapidly suppressed the behavioral seizure score such that within 5–10 min, most animals were in stage 0. There was no mortality associated with the treatment during the 7‐day observation period. We monitored four of the ALLO + PPL + MDZ animals for 3 days. One of these animals had no isolated seizures or SE relapse; two of the animals had sporadic seizure events but no SE relapse; and one animal had sporadic seizure events that in some cases met the definition of SE. The first episode is illustrated in Figure 2C; these SE episodes continued in the next day.

Because animals were hypoactive with MDZ + ALLO + PPL treatment, we sought to better characterize the behavioral impact by assessing LRR in a separate group of eight naive animals not administered DFP. All animals scored positively. In these animals, the average time to LRR was 4.4 ± 0.7 minutes. These animals gained their righting reflex at 83.7 ± 6.1 minutes. The total sleep time, therefore, in this group of animals was 79.3 ± 5.8 minutes. Animals exhibiting LRR remained responsive to tail or toe pinch, indicating that they were sedated but not in a state of general anesthesia.^67^, ^68^

### Simultaneous administration of ALLO and PPL without MDZ is slow and variable to terminate EEG SE

The combination ALLO + PPL was evaluated in five animals. EEG SE eventually ceased in all of the animals. One of the animals stopped in 7 min, while the other four animals had prolonged stopping times (34, 139, 203, and 878 min, respectively), indicating that, in general, the treatment action is slow. There was a statistically significant reduction in the mean EEG RMS amplitude during the 3+ hour epoch (geometric mean ratio = 0.62, 95% CI: 0.12–0.52, *P* = 0.0001) but not in the 1st hour (geometric mean ratio = 0.65, 95% CI: 0.34–1.27, *P* = 0.2065) and the 2nd hour (geometric mean ratio = 0.52, 95% CI: 0.22–1.22, *P* = 0.13), compared with the 40‐min pretreatment time period. The mean RMS amplitude value for this treatment group never reached the baseline value. The mean behavioral seizure score continued to be high in three out of the five animals tested (data not shown). There was no mortality in the 5 h posttreatment observation period. We monitored four of the animals for 2 days. In one, following termination of the initial SE, there were no spikes, isolated seizures, or SE relapse. Another animal exhibited sporadic seizures but no SE relapse. The remaining two animals exhibited sporadic seizures and SE relapses, which occurred initially at 3.6 and 9.7 h following cessation of the initial SE episode.

### Simultaneous administration of MDZ, ALLO, and PPL prevents late SRS

**Figure 5:**
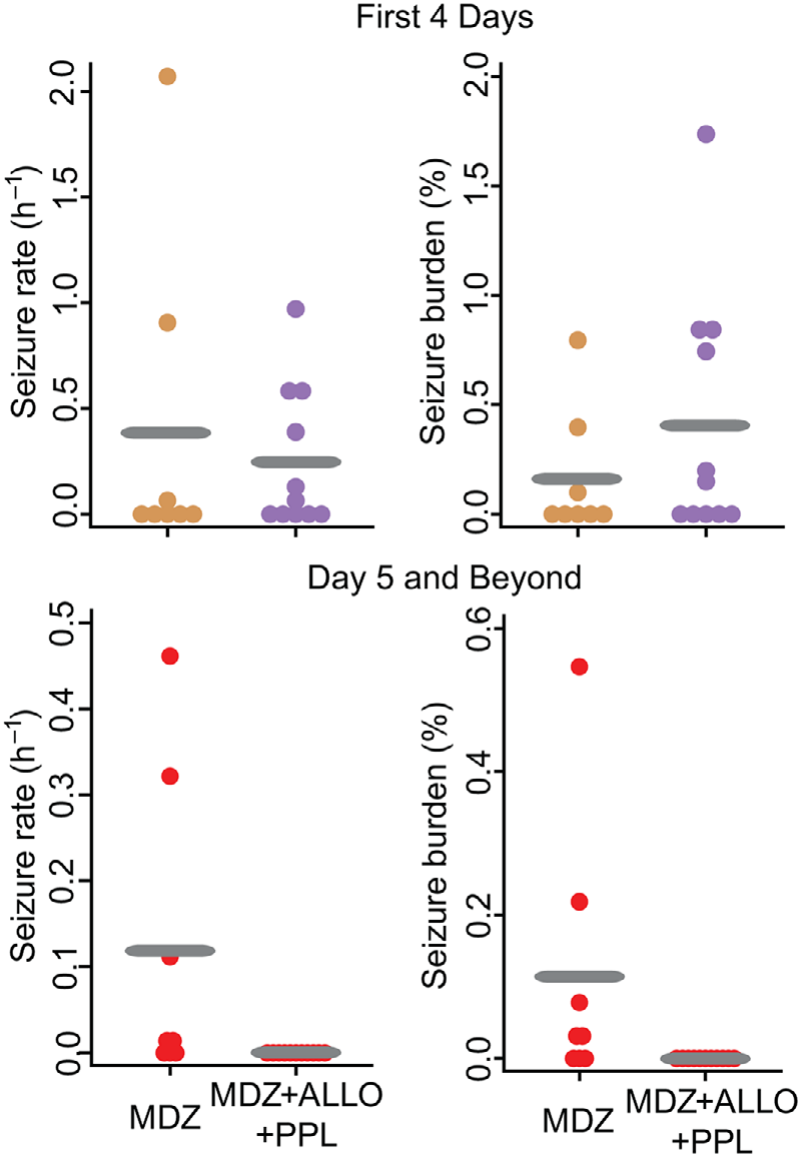
Spontaneous recurrent seizures in DFP animals treated with MDZ or MDZ + ALLO + PPL. Left graphs show seizure rates of individual animals calculated as number of seizures per hour of recording, and right graphs indicate seizure burden calculated as total seconds of seizures per seconds of recording, expressed as a percentage. The top graphs represent data for the first 4 days after DFP; the bottom graphs represent data from day 5 to the end of monitoring. Animals were monitored from 1 to 7.5 months and 3 to 7.5 months in the MDZ (8 animals) and the MDZ + ALLO + PPL (11 animals) groups, respectively; total EEG recording times were 156 ± 28 (mean ± S.E.M.; range 18–444) h and 545 ± 103 (range 53–3883) h, respectively. One animal in the MDZ group died (day 102). Data points represent mean values from each rat analyzed over the entire recording time. Gray bars represent the group means.

Given the powerful effect of the combined therapy MDZ + ALLO + PPL on EEG and behavioral SE, we sought to determine if it would prevent the occurrence of SRS, where the late occurrence of such seizures is an indication of epileptogenesis. We assessed spontaneous EEG seizures in randomly selected recording periods during the first 4 days after DFP treatment and for an extended period after this time. During 4 days after DFP injection (Fig. 5, top panel), animals treated with MDZ and with MDZ + ALLO + PPL exhibited ictal activity. There were no statistically significant differences in seizure rate or seizure burden during this initial recording period according to the Mann–Whitney test. The second time period started on day 5 following DFP and continued for up to 7.5 months (mean = 5.2 ± 0.5 months). Recordings were carried out continuously for 1 week or more in some of the animals. At the end of the monitoring, seizure rate and seizure burden were determined. Six of the eight (75%) MDZ‐treated animals continued to exhibit seizures during the second time period, although three only exhibited a single seizure episode. By contrast, none of the 11 animals in the MDZ + ALLO + PPL group exhibited any seizure episodes. Hence, the two groups differed significantly with respect to seizure rate and seizure burden according to the Mann–Whitney test, with *P* values of 0.001 for each outcome. The Mann–Whitney test does not produce a 95% confidence interval for an effect size parameter estimate corresponding to the relative reduction in seizure incidence between the two groups, but the observed relative reduction was total (100%).

### Animals receiving combination treatments exhibit less neurodegeneration than those receiving MDZ alone

**Figure 6:**
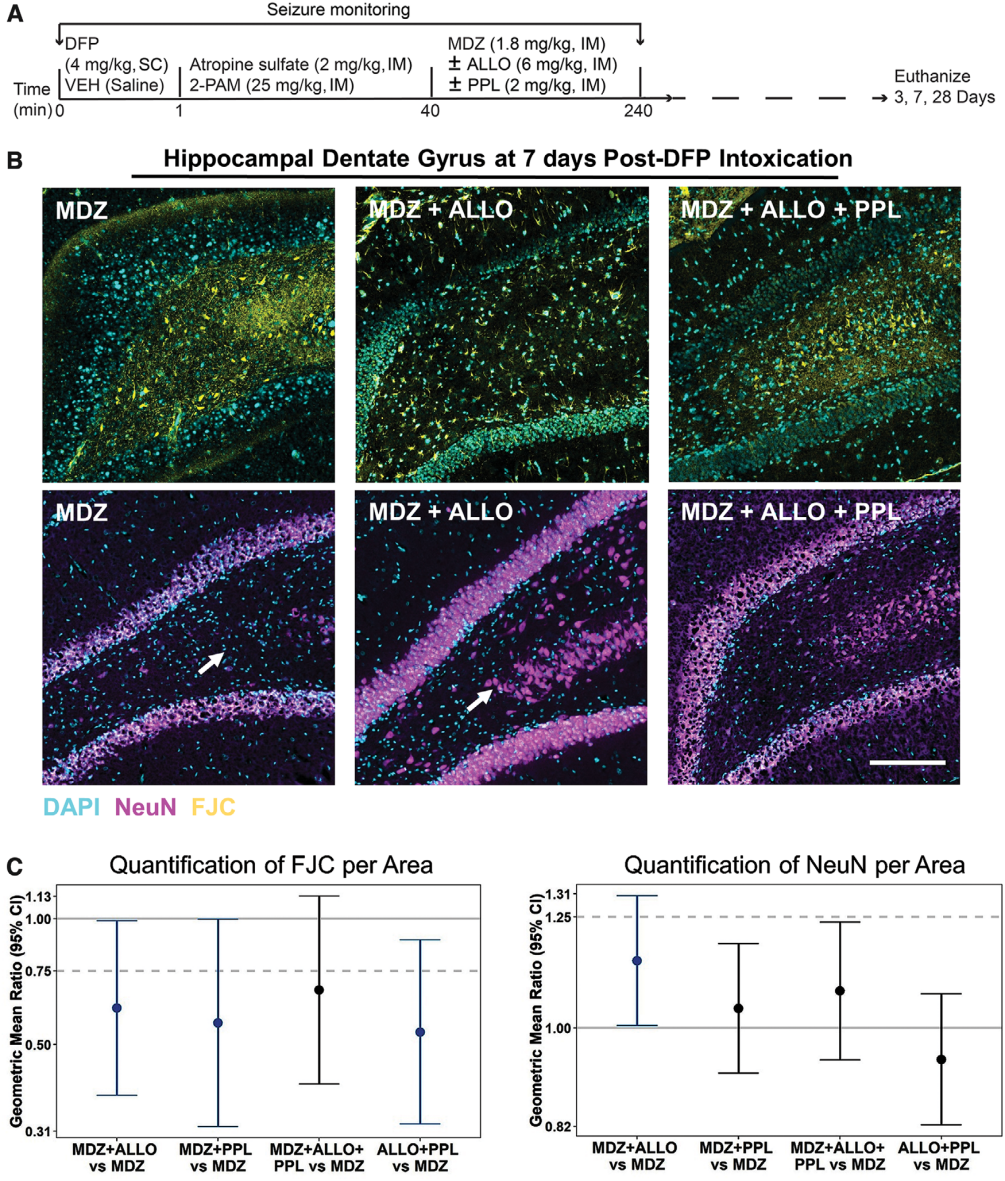
(A) Schematic of the experimental time line. (B) Representative photomicrographs of FluoroJade C (FJC, yellow) and NeuN (purple) staining in the hippocampus at day 7 post‐DFP. Sections were counterstained with DAPI (blue) to label nuclei. Bar = 100 μm (applies to all photomicrographs). Arrows: NeuN^+^ cells in the dentate gyrus protected by combined treatment with MDZ + ALLO. (C) Initial statistical analyses indicated that treatment differences did not vary between brain regions (cortex, CA1, CA3, dentate gyrus, thalamus, amygdala, and piriform cortex) or time postexposure (3, 7, or 28 days), so comparisons between treatments are presented as an overall estimate of the difference between treatments. Summary of geometric mean ratios (and 95% confidence intervals) of mean fluorescence values for the combination treatments to the value for MDZ alone (3–8 animals per group). Confidence intervals that do not encompass a geometric mean ratio of 1 (solid light gray line) are colored blue and indicate a significant difference between groups at a *P* < 0.05. A ratio of 0.75 or below for FJC or 1.25 or above for NeuN indicates 25% improvement compared with MDZ alone.

In a separate series of experiments using the DFP model, we sought to compare the neuroprotective activities of MDZ, MDZ + ALLO, MDZ + PPL, and MDZ + ALLO + PPL. As shown in the schematic of Figure 6A, animals were treated as in the previously described experiments assessing seizure suppression. The animals were then maintained for 3, 7, or 28 days, euthanized, and their brains collected for histological analysis. Automated image acquisition and analysis was used to assess markers of neurodegeneration (FJC staining and NeuN immunoreactivity). At each time point, data were collected across multiple brain regions, including the neocortex; hippocampal CA1, CA3, and dentate gyrus; thalamus; amygdala; and piriform cortex.

In the experiments assessing treatment effects on neurodegeneration, there was insufficient evidence to suggest that treatment differences varied significantly between brain regions or time postexposure (3, 7, or 28 days), so comparisons between treatments were presented as an overall estimate of the difference between treatments; these treatment differences were estimated to be the same across brain regions and time postexposure. To assess whether the combination drug treatments lead to reduced neurodegeneration compared with neurodegeneration occurring in animals receiving MDZ alone, we calculated the ratio with 95% CI of the geometric mean of the values for each combination treatment to the geometric mean for the MDZ‐only group. The results are presented in Figure 6C. The mean ratio was deemed to indicate a significant difference between the combination treatment group and the MDZ‐only group if the 95% CI does not span 1. In the case of FJC staining, we consider a reduction in signal to be an improvement; by contrast, for NeuN immunoreactivity, an increase in signal is considered to be an improvement. We arbitrarily set a difference of 25% as an effect size of interest. MDZ + ALLO, MDZ + PPL, and ALLO + PPL all were associated with a significant reduction in FJC staining compared with MDZ alone, and the mean ratios met the 25% criteria but there was substantial uncertainty, as indicated by the width of the 95% CI, which included 0.75 (Fig. 6B and C). The point estimate for the MDZ + ALLO + PPL group indicated a >25% effect size, but the confidence in the estimate did not meet the criteria for statistical significance. Most treatments did not cause a significant change in NeuN immunoreactivity relative to MDZ alone, except for MDZ + ALLO, which showed a significant increase relative to MDZ alone that met the 25% improvement criteria (Fig. 6B and C).

### Addition of ALLO and PPL to MDZ significantly attenuates astrocytosis and microgliosis but not microglial activation

**Figure 7:**
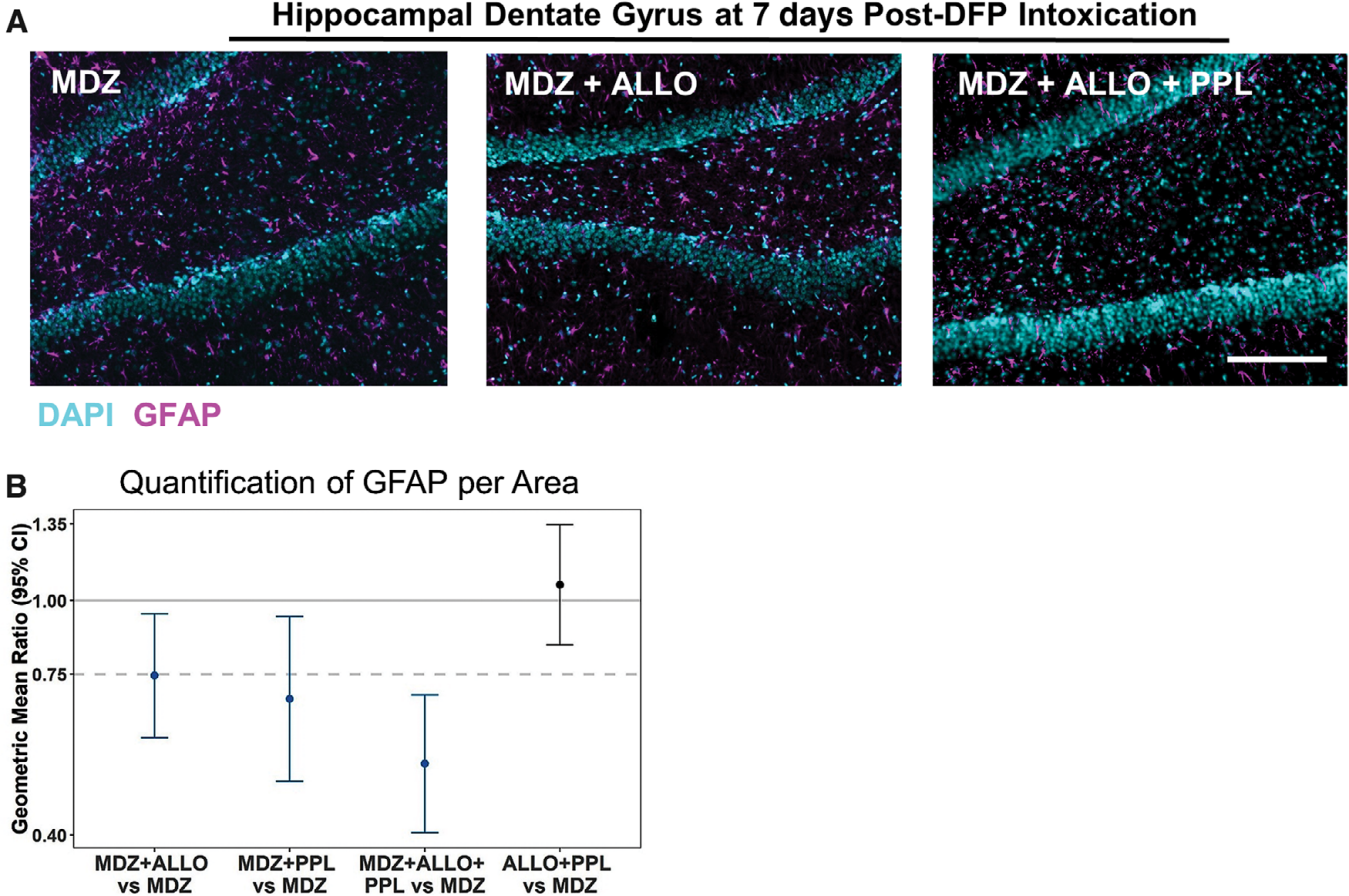
(A) Representative photomicrographs of GFAP (purple) immunoreactivity in the hippocampal dentate gyri of rats at 7 days post‐DFP. Sections were counterstained with DAPI (blue) to label nuclei. Bar = 200 μm (applies to all photomicrographs). (B) Summary of geometric mean ratios (and 95% confidence intervals) of mean fluorescence values for the combination treatments to the value for MDZ alone (5–8 animals per group). Confidence intervals that do not encompass a geometric mean ratio of 1 (solid light gray line) are colored blue and indicate a significant difference between groups at a *P* < 0.05. A ratio of 0.75 (dotted line) or below indicates ≥25% improvement compared with MDZ alone. Estimates are averaged across brain regions (cortex, CA1, CA3, dentate gyrus, thalamus, amygdala, and piriform cortex) and time points (3, 7, and 28 days post‐DFP) because initial statistical analyses indicated no differences in treatment effects between brain regions or between time points.

Sections from the same brains used to assess neurodegeneration were evaluated for treatment effects on astrocytosis and microglial activation at 3, 7, and 28 days following DFP intoxication. Astrogliosis was assessed with GFAP immunostaining. As with FJC and NeuN, no statistically significant differences in treatment effects were found across brain regions or postexposure times, so the estimated treatment differences were expressed as an overall effect, which were the same across brain regions and postexposure times. Representative photomicrographs in Figure 7A show less GFAP staining in the hippocampus after MDZ + ALLO + PPL treatment than MDZ alone. This was confirmed by statistical analysis. Treatment with either MDZ + ALLO, MDZ + PPL, or MDZ + ALLO + PPL was superior to MDZ alone in reducing astrogliosis (Fig. 7B). The combined treatment of MDZ + ALLO + PPL produced the largest relative reduction of approximately 50%. Treatment with ALLO + PPL without MDZ was not significantly better than the treatment with MDZ alone.

**Figure 8:**
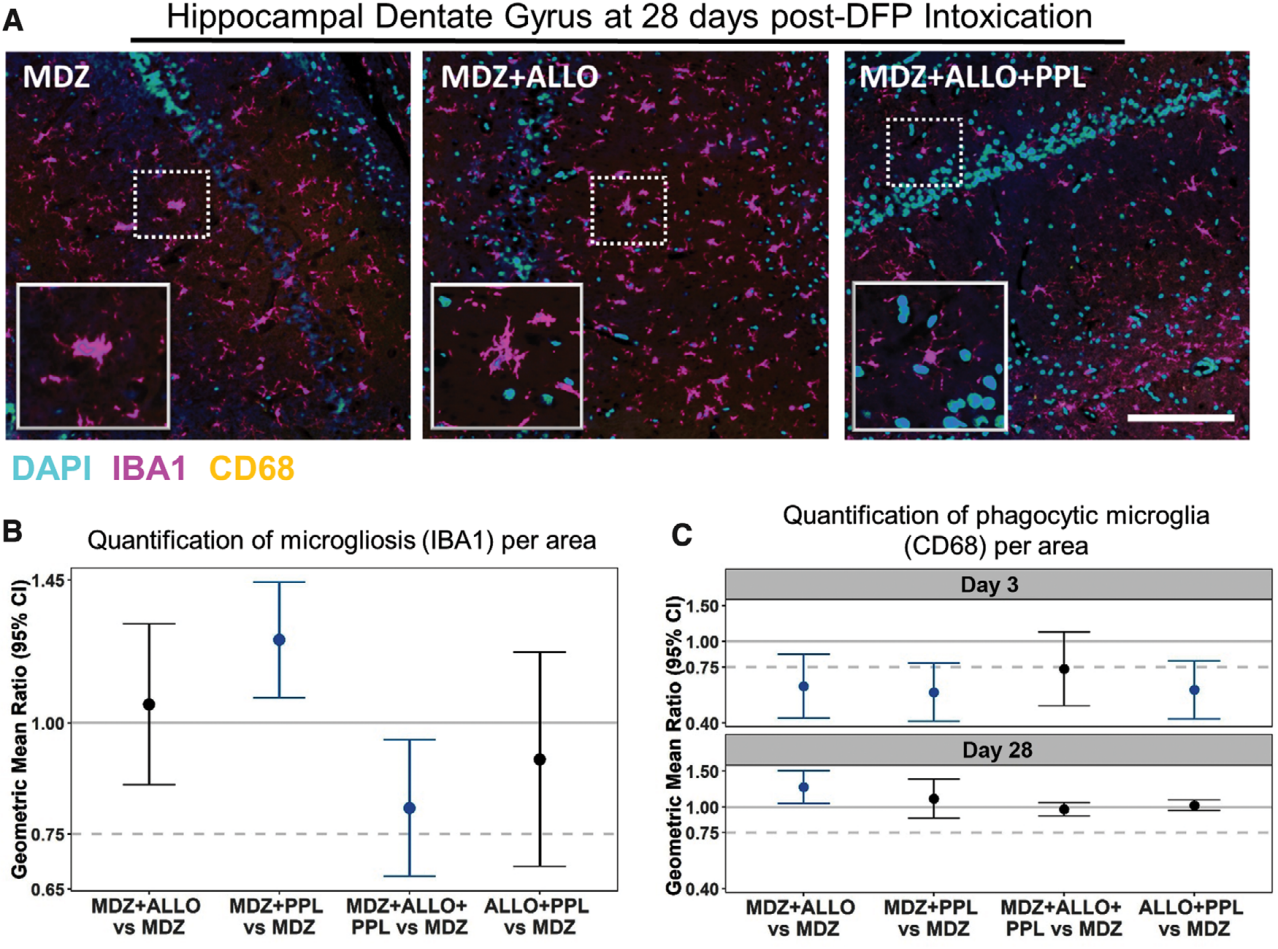
(A) Representative photomicrographs of IBA1 (purple) and CD68 (yellow) immunoreactivity in hippocampal dentate gyri at 28 days post‐DFP. Sections were counterstained with DAPI (blue) to label cell nuclei. Bar = 200 μm (applies to all photomicrographs). Dashed boxes surrounding individual microglia are shown at higher magnification in the insets. (B) Summary of geometric mean ratios (and 95% confidence intervals) of mean IBA1 fluorescence values for the combination treatments to the value for MDZ alone (3–6 animals per group). Estimates are averaged across brain regions (cortex, CA1, CA3, dentate gyrus, thalamus, amygdala, and piriform cortex) and time points (3, 7, and 28 days post‐DFP) because initial statistical analyses indicated no significant differences in treatment effects between brain regions and time points. (C) Summary of geometric mean ratios (and 95% confidence intervals) of percent IBA1 cells colabeled for CD68 averaged across brain regions because initial statistical analyses indicated no significant differences in treatment effects between brain regions. However, significant differences in treatment effects between time points were identified, so estimates of these treatment differences from 3 and 28 days following DFP exposure are shown separately. Confidence intervals that do not encompass a geometric mean ratio of 1 (solid light gray line) are colored blue and indicate a significant difference between groups at a *P* < 0.05. A ratio of 0.75 (dotted line) or below indicates ≥25% improvement compared with MDZ alone.

Microgliosis was assessed as the percent area immunoreactive for IBA‐1, and microglial activation was determined by immunoreactivity for CD68, a marker of phagocytic microglia (Fig. 8A). There was no statistical difference in treatment effects between the brain regions or time points for the IBA‐1 staining, so treatment differences were expressed as an overall effect; again, these treatment differences were estimated to be the same across brain regions and time points. Only the combination treatment of MDZ + ALLO + PPL was demonstrated to be superior to MDZ alone in reducing microgliosis (Fig. 8B). The effect of treatment on number of phagocytic microglia, as detected by CD68, differed by time postexposure, but not brain region. Therefore, treatment differences were expressed by time point and estimated to be the same across brain region. In particular, the number of phagocytic microglia was significantly reduced with MDZ + ALLO, MDZ + PPL, and ALLO + PPL compared with MDZ alone at 3 days post‐DFP exposure, but the group receiving MDZ + ALLO + PPL did not reach significance (Fig. 8C). There was no significant improvement (decrease in the number of phagocytic microglia) in any of the treatment groups and MDZ alone at 28 days following DFP treatment. At 28 days following DFP exposure, the MDZ + ALLO treatment group exhibited a significant increase in phagocytic microglia.

## Discussion

Treatment of rats with DFP rapidly induced continuous electrographic and behavioral SE, with a mean time to onset of 6–9 min and persistence of more than 5 hours. The International League Against Epilepsy defines SE as continuous seizure activity that lasts for 5 min or more and recognizes that if seizures persist for more than 30 min, there is a high risk of long‐term consequences,^69^ such as neurodegeneration, epileptogenesis, and cognitive decline.^7^, ^64^, ^70^, ^71^, ^72^, ^73^ Here, we administered treatments at 40 min, a time in the evolution of SE where there is irreversible morbidity. SE in rats receiving 4 mg/kg DFP along with atropine and 2‐PAM usually survive although some animals do succumb, which was the case for 25% of the animals in our vehicle treatment group. This mortality rate is comparable with the 16% rate observed by Pouliot and colleagues.^70^

In this study, we examined single doses of MDZ alone and in combination with single doses of ALLO and/or PPL as treatments for DFP SE. We chose to limit the study to single doses and explore the various possible adjunctive combinations; we plan to investigate other doses in later studies. The dose of MDZ (1.8 mg/kg) was equivalent to the maximum recommended midazolam dose for the emergency treatment of nerve agent seizures in adults. The doses of ALLO and PPL, 6 and 2 mg/kg, respectively, were based on prior studies reported in the literature and preliminary studies with intramuscular dosing in the pentylenetetrazol model. Subcutaneous ALLO at a similar dose (5 mg/kg) exhibited robust antiseizure activity in the rat pentylenetetrazol seizure model.^74^ ALLO at doses similar to that used here has also been reported to prevent pilocarpine‐induced SE in mice^19^ and to reduce the duration of kainate seizures in rats.^75^ However, in preliminary experiments in two rats, we found that by itself 6 mg/kg intramuscular ALLO does not abort DFP‐induced SE (data not shown). PPL has demonstrated antiseizure activity in several rat seizure models when administered by various routes,^76^ including at 2 mg/kg with intraperitoneal administration in a rapid kindling model,^40^ at 2–3 mg/kg by intraperitoneal administration in mouse models of tetramethylenedisulfotetramine‐induced seizures and SE,^77^, ^78^ and at 2–6 mg/kg with intravenous administration in a lithium‐pilocarpine SE model.^36^, ^79^ In preliminary experiments, we found that PPL at a dose of 2 mg/kg intramuscularly (30 min pretreatment time) increased the thresholds for myoclonic jerks, clonus, and tonic extension in the mouse timed intravenous pentylenetetrazol seizure threshold test (data not shown).

We can only draw conclusions regarding the specific doses used. Higher doses, to the extent they are tolerated, could produce different or more robust responses. With this important limitation in mind, we have confirmed in this study that MDZ fails to terminate DFP‐induced electrographic or behavioral seizures^4^, ^14^, ^80^ although it does modestly suppress EEG RMS amplitude in relation to vehicle treatment as recently reported by Spampanato *et al*.^81^ We note that in contrast to this latter study, we did not pretreat animals with pyridostigmine bromide because we have found it to have no effect on seizure behavior or 24 h survival of rats intoxicated with DFP.^82^

The combination MDZ + ALLO caused a rapid and persistent suppression of EEG amplitude and behavioral seizure score in eight of 10 animals but did not terminate SE and eliminate behavioral seizures in the remaining two animals. The speed of this combination likely results from the speed of absorption and distribution to the brain of intramuscular MDZ (plasma *T*_max_ = 2.3 min;^83^ treatment latency = 9–13 min^51^) and also the speed of intramuscular ALLO (plasma *T*_max_ = 1 min; brain *T*_max_ = 10 minutes^20^). The time to seizure termination with MDZ + ALLO treatment (mean latency of 9 min) is compatible with the pharmacokinetics of each component of the combination. Both MDZ and ALLO have relatively fast elimination rates (plasma *T*_1/2_ = 20.7 min for MDZ;^83^ plasma *T*_1/2_ = 16 min for ALLO^20^, ^21^). Nevertheless, MDZ + ALLO treatment caused persistent >3‐h seizure suppression relative to baseline. We do note, however, that while comparisons with the vehicle and MDZ groups showed the RMS EEG amplitude to be reduced more in the MDZ + ALLO group in all three epochs, the comparisons were not statistically significant for the 3+ hour epoch. Although plasma levels of MDZ and ALLO fall rapidly, both compounds are retained to some extent in the brain (<10–20% of peak levels at 120 minutes^20^, ^83^). The persistent low level brain exposures may contribute to the long duration seizure suppression observed in many animals. However, some animals in the MDZ + ALLO group exhibited what appeared to be isolated seizures following SE termination and in one animal, the EEG activity met our criteria for relapse of SE (Fig. 3C). In the early period following SE termination, some of the animals treated with MDZ + ALLO also exhibited high‐amplitude phenomena on the time scale of 1–2.5 s with an otherwise low‐amplitude background. These events may not be epileptic in nature but rather could represent burst suppression.^84^ Burst suppression is a well‐recognized EEG phenomenon that occurs with general anesthetics that act by enhancing GABA_A_ receptors. The antiseizure effect of both MDZ and ALLO is through this mechanism and both can cause burst suppression at anesthetic doses.^85^, ^86^, ^87^

MDZ + PPL had a slow action but over time suppressed the EEG amplitude to below baseline and eliminated behavioral seizures. In studies with oral administration in the rat, PPL has relatively slow absorption and distribution kinetics (oral plasma *T*_max_ = 30–60 min^76^), and exhibits slow brain entry (oral brain *T*_max_ = 90 minutes^88^). Although pharmacokinetic data for intramuscular PPL are not available, the slow onset with MDZ + PPL likely has a pharmacokinetic basis.

The three‐drug combination MDZ + ALLO + PPL rapidly suppressed EEG amplitude and largely prevented late rhythmic spike activity. At the same time, it rapidly and consistently eliminated behavioral seizures. We, therefore, conclude that in terms of ability to confer rapid and persistent seizure suppression, it is superior to the other combinations tested, perhaps because it invokes multiple antiseizure mechanisms but also because the slow pharmacokinetics of PPL compensates for the rapid elimination of MDZ and ALLO. As was the case with MDZ + ALLO, burst suppression‐like events were noted. Intermittent seizures and brief seizure relapse were observed in the 3‐day period after treatment with the three‐drug combination, likely due to the persistence of brain cholinesterase inhibition.^89^, ^90^ We do not consider the occurrence of such early spontaneous seizures to be an indication of epileptogenesis. However, the animals in the MDZ‐only group exhibited spontaneous seizures that persisted for up to 7.5 months and clearly had become epileptic. It is noteworthy that the combination MDZ + ALLO + PPL eliminated the occurrence of late spontaneous seizures. While we did not monitor animals continuously, we conducted recordings from the MDZ and MDZ + ALLO + PPL contemporaneously, with a longer total recording time in the latter group. Therefore, while we cannot completely exclude the possibility that animals in the MDZ + ALLO + PPL group may have had seizures at the times they were not monitored, they clearly had fewer seizures than the MDZ group, strongly supporting an antiepileptogenic effect of the combination treatment. The antiepileptogenic action does not imply a mechanism independent of the strong SE suppression provided by the treatment, since SE is the proximate trigger for epileptogenesis. We did not evaluate whether the two‐drug combinations are as effective as the three‐drug combination in preventing the occurrence of late seizures.

Inclusion of MDZ is required for high efficacy and a consistent suppression of SE. While the combination ALLO + PPL did stop SE in most animals, in one animal (20%), it did not, and there was an overall high (30%) mortality comparable with that of vehicle (25%). By contrast, there was no mortality in animals receiving MDZ + ALLO or MDZ + ALLO + PPL. Although MDZ + ALLO + PPL performed best in terminating SE, it was associated with greater sedation than the other treatments and caused prolonged LRR (>1 h) but did not suppress responses to noxious stimulation. The sedation was not associated with cardiorespiratory compromise and all animals fully recovered. Nevertheless, the extent to which such prolonged depression of consciousness would be tolerated in the emergency treatment of nerve agent intoxication requires consideration.

The dual combination of MDZ with ALLO or PPL conferred enhanced neuroprotection as assessed by FJC staining when compared with MDZ alone. The geometric mean ratio for FJC was reduced by >25% so that these treatment regimens met the arbitrary criterion of substantial efficacy. By contrast, treatment with MDZ + ALLO + PPL, the combination that performed best in terms of seizure termination, did not exhibit greater neuroprotection. While the statistical criterion for greater efficacy than MDZ alone was not met with MDZ + ALLO + PPL, the point estimate did exceed the arbitrary threshold for substantial efficacy. Considering the data as a whole, it is reasonable to conclude that all of the treatment combinations confer neuroprotection greater than MDZ alone and that none of the combinations is clearly superior. Unlike FJC, NeuN, which labels neuronal nuclei, does not assess the degree of short‐term neuronal loss. Rather, loss of NeuN immunostaining corresponds with neurodegeneration occurring several weeks after brain insults, including OP seizures.^55^, ^91^ Thus, in previous studies of the DFP SE model, profound reductions in NeuN staining were observed 28 days following DFP treatment in brain regions corresponding with areas of neuronal cell loss and neuroinflammation.^6^ In the present study, most of the combination treatments were associated with a modest increase in NeuN immunostaining compared with MDZ alone, but none met the 25% increase criterion and only the combination of MDZ + ALLO was significantly superior to MDZ alone, suggesting that improved seizure termination does not translate into a robust improvement in late neurodegeneration.

It has previously been shown that DFP‐induced SE is associated with persistent increases in GFAP protein expression in multiple rat brain regions.^6^, ^7^, ^55^, ^64^ Most of the treatment combinations restrained DFP‐induced GFAP expression more than MDZ alone. The triple combination MDZ + ALLO + PPL was superior to the other combinations and the 95% CI of the geometric mean ratio fell below 0.75, indicating that the magnitude of the reduction met the arbitrary criterion for substantial efficacy with statistical significance. Interestingly, however, ALLO + PPL was no more effective than MDZ, which is perhaps not surprising given its inconsistency in suppressing seizures. The lack of effect on the GFAP marker is not consistent with the significantly reduced FJC staining with ALLO + PPL relative to MDZ alone.

The effect of the combined treatments on microgliosis was mixed. MDZ + ALLO and ALLO + PPL did not reduce microgliosis more than MDZ alone. By contrast, MDZ + PPL significantly increased microgliosis, which is in line with its ineffectiveness in suppressing seizures. Only the triple combination of MDZ + ALLO + PPL was significantly more effective than MDZ alone in decreasing microgliosis. Thus, it was surprising that treatment with MDZ + ALLO + PPL was not more effective than MDZ alone in mitigating microglial cell activation, as indicated by expression of CD68, a biomarker of phagocytosis. The dual combinations of MDZ + ALLO, MDZ + PPL, and ALLO + PPL were significantly more effective than MDZ alone at 3 days but not 28 days following DFP exposure. Overall, the present results are consistent with previous studies of OP‐induced SE, in which it was concluded that neurodegeneration and neuroinflammation are mediated by independent mechanisms,^92^ and that seizure severity and duration are not the only variables driving neuronal cell loss.^90^

In conclusion, our results confirm that MDZ at a dose equivalent to that recommended for the treatment of OP nerve agent seizures does not terminate DFP‐induced SE in a rat model when administered 40 min after seizure onset. However, when ALLO or ALLO + PPL are administered with the same dose of MDZ, SE is generally terminated and there is reduced neurodegeneration. We have further shown that adjunctive ALLO + PPL prevents the late occurrence of SRS that are present in animals treated with MDZ alone. Overall, improved seizure control was, in general, associated with neuroprotection and reductions in at least some aspects of neuroinflammation. The lack of a direct relationship between seizure control and these late outcomes raises the possibility that other factors may be relevant. MDZ and ALLO are both positive allosteric modulators of GABA_A_ receptors but MDZ only acts on GABA_A_ receptor isoforms expressed at synapses, whereas ALLO acts on all GABA_A_ receptor subunit combinations, including those expressed extrasynaptically that mediate tonic inhibition.^93^ We and others have reported synergistic interactions between benzodiazepines with ALLO in *in vitro* systems as well as in *in vivo* seizure models.^94^, ^95^, ^96^ We now extend the evidence of synergism to an OP seizure model. The present study demonstrates that the addition of PPL further enhances the activity of the MDZ + ALLO combination, but the sedation produced by the three‐drug combination may limit its utility. Given the proposal that excessive glutamate‐mediated excitation contributes to seizure generation in the later stages of OP poisoning, there is a strong theoretical reason to believe that an AMPA receptor antagonist, such as PPL, could be useful in the treatment of refractory OP seizures.^26^ However, our results suggest that PPL does not have the appropriate pharmacokinetic properties to be useful as an adjunctive agent by itself although it could be a useful component of a combination regimen with ALLO or another agent that acts through effects on GABA‐mediated inhibition.

## Author contributions

A.D. conceived the study, conducted electrophysiological and behavioral experiments, analyzed and interpreted the data, wrote the first draft, and revised the manuscript. D.A.B., M.G., Y.‐H.T., E.G., J.C., and J.V. conducted histology experiments and contributed to the first draft of the manuscript. D.J.T., D.J.H., and N.S. designed and conducted statistical analyses. P.J.L. conceived the study, analyzed and interpreted the histology data, and edited the manuscript. M.A.R. conceived the study, analyzed and interpreted the data, and wrote the final manuscript. All authors approved the final version of the submitted manuscript.

## Competing interests

A.D., P.J.L., and M.A.R. are named inventors of patents and patent applications assigned to the Regents of the University of California that are relevant to the work described here. M.A.R. has served as a consultant to Eisai, Sage Therapeutics, and Marinus Pharmaceuticals. All other authors declare no competing interests.

## References

[1] Karanth, S. , J. Liu , A. Ray & C. Pope . 2007 Comparative *in vivo* effects of parathion on striatal acetylcholine accumulation in adult and aged rats. Toxicology 239: 167–179. 17707571 10.1016/j.tox.2007.07.004

[2] Waheed, S. , A. Sabeen & N. Ullah Khan . 2014 New onset refractory status epilepticus as an unusual presentation of a suspected organophosphate poisoning. Case Rep. Emerg. Med. 2014: 676358. 25580311 10.1155/2014/676358 PMC4281438

[3] Myhrer, T. 2007 Neuronal structures involved in the induction and propagation of seizures caused by nerve agents: implications for medical treatment. Toxicology 239: 1–14. 17689166 10.1016/j.tox.2007.06.099

[4] Todorovic, M.S. , M.L. Cowan , C.A. Balint , *et al* 2012 Characterization of status epilepticus induced by two organophosphates in rats. Epilepsy Res. 101: 268–276. 22578704 10.1016/j.eplepsyres.2012.04.014 PMC3419801

[5] McDonough, J.H., Jr. & T.M. Shih . 1997 Neuropharmacological mechanisms of nerve agent‐induced seizure and neuropathology. Neurosci. Biobehav. Rev. 21: 559–579. 9353792 10.1016/s0149-7634(96)00050-4

[6] Sisó, S. , B.A. Hobson , D.J. Harvey , *et al* 2017 Editor's Highlight: spatiotemporal progression and remission of lesions in the rat brain following acute intoxication with diisopropylfluorophosphate. Toxicol. Sci. 157: 330–341. 28329845 10.1093/toxsci/kfx048 PMC6070115

[7] Guignet, M. , K. Dhakal , B.M. Flannery , *et al* 2020 Persistent behavior deficits, neuroinflammation, and oxidative stress in a rat model of acute organophosphate intoxication. Neurobiol. Dis. 133: 104431. 30905768 10.1016/j.nbd.2019.03.019 PMC6754818

[8] de Araujo Furtado, M. , L.A. Lumley , C. Robison , *et al* 2010 Spontaneous recurrent seizures after status epilepticus induced by soman in Sprague–Dawley rats. Epilepsia 51: 1503–1510. 20067510 10.1111/j.1528-1167.2009.02478.x

[9] Chuang, C.S. , K.W. Yang , C.M. Yen , *et al* 2019 Risk of seizures in patients with organophosphate poisoning: a nationwide population‐based study. Int. J. Environ. Res. Public Health 16: 3147. 10.3390/ijerph16173147 PMC6747140 31470499

[10] Newmark, J. 2019 Therapy for acute nerve agent poisoning: an update. Neurol. Clin. Pract. 9: 337–342. 31583189 10.1212/CPJ.0000000000000641 PMC6745742

[11] McDonough, J.H. , J.D. McMonagle & T.M. Shih . 2010 Time‐dependent reduction in the anticonvulsant effectiveness of diazepam against soman‐induced seizures in guinea pigs. Drug Chem. Toxicol. 33: 279–283. 20429808 10.3109/01480540903483417

[12] Shrot, S. , E. Ramaty , Y. Biala , *et al* 2014 Prevention of organophosphate‐induced chronic epilepsy by early benzodiazepine treatment. Toxicology 323: 19–25. 24881594 10.1016/j.tox.2014.05.010

[13] Jett, D.A. & S.M. Spriggs . 2020 Translational research on chemical nerve agents. Neurobiol. Dis. 133: 104335. 30468862 10.1016/j.nbd.2018.11.020

[14] Wu, X. , R. Kuruba & D.S. Reddy . 2018 Midazolam‐resistant seizures and brain injury after acute intoxication of diisopropylfluorophosphate, an organophosphate pesticide and surrogate for nerve agents. J. Pharmacol. Exp. Ther. 367: 302–321. 30115757 10.1124/jpet.117.247106 PMC6193253

[15] Goodkin, H.P. , S. Joshi , Z. Mtchedlishvili , *et al* 2008 Subunit‐specific trafficking of GABA_A_ receptors during status epilepticus. J. Neurosci. 28: 2527–2538. 18322097 10.1523/JNEUROSCI.3426-07.2008 PMC2880323

[16] Wasterlain, C.G. , H. Liu , D.E. Naylor , *et al* 2009 Molecular basis of self‐sustaining seizures and pharmacoresistance during status epilepticus: the receptor trafficking hypothesis revisited. Epilepsia 50 (Suppl. 12): 16–18. 10.1111/j.1528-1167.2009.02375.x 19941513

[17] Rogawski, M.A. , C.M. Loya , K. Reddy , *et al* 2013 Neuroactive steroids for the treatment of status epilepticus. Epilepsia 54 (Suppl. 6): 93–98. 24001085 10.1111/epi.12289 PMC3772544

[18] Reddy, D.S. & M.A. Rogawski . 2012 Neurosteroids — endogenous regulators of seizure susceptibility and role in the treatment of epilepsy In: Jasper's Basic Mechanisms of the Epilepsies [Internet]. 4th ed. Noebels J.L., Avoli M., Rogawski M.A., *et al*, Eds. Bethesda, MD: National Center for Biotechnology Information (US). 22787590

[19] Kokate, T.G. , A.L. Cohen , E. Karp & M.A. Rogawski . 1996 Neuroactive steroids protect against pilocarpine‐ and kainic acid‐induced limbic seizures and status epilepticus in mice. Neuropharmacology 35: 1049–1056. 9121607 10.1016/s0028-3908(96)00021-4

[20] Zolkowska, D. , C.Y. Wu & M.A. Rogawski . 2018 Intramuscular allopregnanolone and ganaxolone in a mouse model of treatment‐resistant status epilepticus. Epilepsia 59(Suppl. 2): 220–227. 29453777 10.1111/epi.13999 PMC6910080

[21] Saporito, M.S. , J.A. Gruner , A. DiCamillo , *et al* 2019 Intravenously administered ganaxolone blocks diazepam‐resistant lithium‐pilocarpine‐induced status epilepticus in rats: comparison with allopregnanolone. J. Pharmacol. Exp. Ther. 368: 326–337. 30552296 10.1124/jpet.118.252155

[22] Barker, B.S. , J. Spampanato , H.S. McCarren , *et al* 2020 Screening for efficacious anticonvulsants and neuroprotectants in delayed treatment models of organophosphate‐induced status epilepticus. Neuroscience 425: 280–300. 31783100 10.1016/j.neuroscience.2019.11.020 PMC6935402

[23] Biagini, G. , E. Baldelli , D. Longo , *et al* 2006 Endogenous neurosteroids modulate epileptogenesis in a model of temporal lobe epilepsy. Exp. Neurol. 201: 519–524. 16780839 10.1016/j.expneurol.2006.04.029

[24] Biagini, G. , D. Longo , E. Baldelli , *et al* 2009 Neurosteroids and epileptogenesis in the pilocarpine model: evidence for a relationship between P450scc induction and length of the latent period. Epilepsia 50 (Suppl. 1): 53–58. 19125849 10.1111/j.1528-1167.2008.01971.x PMC4873280

[25] Tattersall, J. 2009 Seizure activity post organophosphate exposure. Front. Biosci. (Landmark Ed.) 14: 3688–3711. 19273303 10.2741/3481

[26] Aroniadou‐Anderjaska, V. , T.H. Figueiredo , J.P. Apland & M.F. Braga . 2020 Targeting the glutamatergic system to counteract organophosphate poisoning: a novel therapeutic strategy. Neurobiol. Dis. 133: 104406. 30798006 10.1016/j.nbd.2019.02.017

[27] Dorandeu, F. , L. Barbier , F. Dhote , *et al* 2013 Ketamine combinations for the field treatment of soman‐induced self‐sustaining status epilepticus. Review of current data and perspectives. Chem. Biol. Interact. 203: 154–159. 23044489 10.1016/j.cbi.2012.09.013

[28] Lumley, L.A. , F. Rossetti , M. de Araujo Furtado , *et al* 2019 Dataset of EEG power integral, spontaneous recurrent seizure and behavioral responses following combination drug therapy in soman‐exposed rats. Data Brief 27: 104629. 31687442 10.1016/j.dib.2019.104629 PMC6820070

[29] Niquet, J. , L. Lumley , R. Baldwin , *et al* 2020 Rational polytherapy in the treatment of cholinergic seizures. Neurobiol. Dis. 133: 104537. 31454548 10.1016/j.nbd.2019.104537

[30] Fritsch, B. , J. Reis , M. Gasior , *et al* 2014 Role of GluK1 kainate receptors in seizures, epileptic discharges, and epileptogenesis. J. Neurosci. 34: 5765–5775. 24760837 10.1523/JNEUROSCI.5307-13.2014 PMC3996208

[31] Höfler, J. & E. Trinka . 2018 Intravenous ketamine in status epilepticus. Epilepsia 59(Suppl. 2): 198–206. 30146731 10.1111/epi.14480

[32] Dorandeu, F. , P. Carpentier , D. Baubichon , *et al* 2005 Efficacy of the ketamine–atropine combination in the delayed treatment of soman‐induced status epilepticus. Brain Res. 1051: 164–175. 16005443 10.1016/j.brainres.2005.06.013

[33] Lewine, J.D. , W. Weber , A. Gigliotti , *et al* 2018 Addition of ketamine to standard‐of‐care countermeasures for acute organophosphate poisoning improves neurobiological outcomes. Neurotoxicology 69: 37–46. 30172622 10.1016/j.neuro.2018.08.011

[34] Rogawski, M.A. 2013 AMPA receptors as a molecular target in epilepsy therapy. Acta Neurol. Scand. Suppl. 9–18. 10.1111/ane.12099. 23480151 PMC4506648

[35] Rogawski, M.A. 2017 Perampanel (Chapter 59) In: Pellock's Pediatric Epilepsy: Diagnosis and Therapy. 4th ed Pellock J.M., Nordli D.R. Jr., Sankar R. & Wheless J.W., Eds.: 861–872. New York: Demos Medical Publishing.

[36] Wu, T. , K. Ido , Y. Osada , *et al* 2017 The neuroprotective effect of perampanel in lithium‐pilocarpine rat seizure model. Epilepsy Res. 137: 152–158. 28624183 10.1016/j.eplepsyres.2017.06.002

[37] Brigo, F. , S. Lattanzi , A. Rohracher , *et al* 2018 Perampanel in the treatment of status epilepticus: a systematic review of the literature. Epilepsy Behav. 86: 179–186. 30076046 10.1016/j.yebeh.2018.07.004

[38] Hocker, S. 2019 Perampanel for refractory status epilepticus… another tool in the armamentarium. Neurocrit. Care 31: 30–31. 31147899 10.1007/s12028-019-00738-z

[39] Strzelczyk, A. , S. Knake , R. Kälviäinen , *et al* 2019 Perampanel for treatment of status epilepticus in Austria, Finland, Germany, and Spain. Acta Neurol. Scand. 139: 369–376. 30613951 10.1111/ane.13061 PMC6590284

[40] Dupuis, N. , J. Enderlin , J. Thomas , *et al* 2017 Anti‐ictogenic and antiepileptogenic properties of perampanel in mature and immature rats. Epilepsia 58: 1985–1992. 28850671 10.1111/epi.13894

[41] Mohammad, H. , S. Sekar , Z. Wei , *et al* 2019 Perampanel but not amantadine prevents behavioral alterations and epileptogenesis in pilocarpine rat model of status epilepticus. Mol. Neurobiol. 56: 2508–2523. 30039334 10.1007/s12035-018-1230-6

[42] Lallement, G. , I. Pernot‐Marino , D. Baubichon , *et al* 1994 Modulation of soman‐induced neuropathology with an anticonvulsant regimen. Neuroreport 5: 2265–2268. 7881042 10.1097/00001756-199411000-00015

[43] McDonough, J.H., Jr. , L.W. Dochterman , C.D. Smith & T.M. Shih . 1995 Protection against nerve agent‐induced neuropathology, but not cardiac pathology, is associated with the anticonvulsant action of drug treatment. Neurotoxicology 16: 123–132. 7603632

[44] Siso, S. , B.A. Hobson , D.J. Harvey , *et al* 2017 Spatiotemporal progression and remission of lesions in the rat brain following acute intoxication with diisopropylfluorophosphate. Toxicol. Sci. 157: 330–341. 28329845 10.1093/toxsci/kfx048 PMC6070115

[45] Shih, T.M. 1990 Anticonvulsant effects of diazepam and MK‐801 in soman poisoning. Epilepsy Res. 7: 105–116. 2289469 10.1016/0920-1211(90)90095-d

[46] Gilat, E. , T. Kadar , A. Levy , *et al* 2005 Anticonvulsant treatment of sarin‐induced seizures with nasal midazolam: an electrographic, behavioral, and histological study in freely moving rats. Toxicol. Appl. Pharmacol. 209: 74–85. 16271623 10.1016/j.taap.2005.03.007

[47] Weissman, B.A. & L. Raveh . 2008 Therapy against organophosphate poisoning: the importance of anticholinergic drugs with antiglutamatergic properties. Toxicol. Appl. Pharmacol. 232: 351–358. 18680758 10.1016/j.taap.2008.07.005

[48] Masson, P. 2011 Evolution of and perspectives on therapeutic approaches to nerve agent poisoning. Toxicol. Lett. 206: 5–13. 21524695 10.1016/j.toxlet.2011.04.006

[49] Hobson, B.A. , S. Siso , D.J. Rowland , *et al* 2017 Magnetic resonance imaging reveals progressive brain injury in rats acutely intoxicated with diisopropylfluorophosphate. Toxicol. Sci. 157: 342–353. 28329842 10.1093/toxsci/kfx049 PMC5458789

[50] McDonough, J.H., Jr. , J. McMonagle , T. Copeland , *et al* 1999 Comparative evaluation of benzodiazepines for control of soman‐induced seizures. Arch. Toxicol. 73: 473–478. 10650919 10.1007/s002040050637

[51] de Araujo Furtado, M. , F. Rossetti , S. Chanda & D. Yourick . 2012 Exposure to nerve agents: from status epilepticus to neuroinflammation, brain damage, neurogenesis and epilepsy. Neurotoxicology 33: 1476–1490. 23000013 10.1016/j.neuro.2012.09.001

[52] Jett, D.A. 2016 The NIH Countermeasures Against Chemical Threats Program: overview and special challenges. Ann. N.Y. Acad. Sci. 1374: 5–9. 27398820 10.1111/nyas.13179 PMC4943675

[53] Gao, J. , S.X. Naughton , H. Wulff , *et al* 2016 Diisopropylfluorophosphate impairs the transport of membrane‐bound organelles in rat cortical axons. J. Pharmacol. Exp. Ther. 356: 645–655. 26718240 10.1124/jpet.115.230839 PMC4767389

[54] Heiss, D.R. , D.W. Zehnder 2nd , D.A. Jett , *et al* 2016 Synthesis and storage stability of diisopropylfluorophosphate. J. Chem. 2016: 3190891. 28856029 10.1155/2016/3190891 PMC5573265

[55] Li, Y. , P.J. Lein , C. Liu , *et al* 2011 Spatiotemporal pattern of neuronal injury induced by DFP in rats: a model for delayed neuronal cell death following acute OP intoxication. Toxicol. Appl. Pharmacol. 253: 261–269. 21513723 10.1016/j.taap.2011.03.026 PMC3108263

[56] Deshpande, L.S. , D.S. Carter , R.E. Blair & R.J. DeLorenzo . 2010 Development of a prolonged calcium plateau in hippocampal neurons in rats surviving status epilepticus induced by the organophosphate diisopropylfluorophosphate. Toxicol. Sci. 116: 623–631. 20498005 10.1093/toxsci/kfq157 PMC2905411

[57] Dhir, A. , D. Zolkowska & M.A. Rogawski . 2013 Seizure protection by intrapulmonary delivery of midazolam in mice. Neuropharmacology 73: 425–431. 23774136 10.1016/j.neuropharm.2013.06.002

[58] Kadriu, B. , A. Guidotti , E. Costa , *et al* 2011 Acute imidazenil treatment after the onset of DFP‐induced seizure is more effective and longer lasting than midazolam at preventing seizure activity and brain neuropathology. Toxicol. Sci. 120: 136–145. 21097996 10.1093/toxsci/kfq356

[59] Reddy, D.S. , D. Perumal , V. Golub , *et al* 2020 Phenobarbital as alternate anticonvulsant for organophosphate‐induced benzodiazepine‐refractory status epilepticus and neuronal injury. Epilepsia Open 5: 198–212. 32524045 10.1002/epi4.12389 PMC7278559

[60] Libenson, M.H. 2010 Practical Approach to Electroencephalography. Philadelphia, PA: Elsevier/Saunders.

[61] Greene, B.R. , S. Faul , W.P. Marnane , *et al* 2008 A comparison of quantitative EEG features for neonatal seizure detection. Clin. Neurophysiol. 119: 1248–1261. 18381249 10.1016/j.clinph.2008.02.001

[62] Fergus, P. , D. Hignett , A. Hussain , *et al* 2015 Automatic epileptic seizure detection using scalp EEG and advanced artificial intelligence techniques. Biomed. Res. Int. 2015: 986736. 25710040 10.1155/2015/986736 PMC4325968

[63] Nissinen, J. , T. Halonen , E. Koivisto & A. Pitkänen . 2000 A new model of chronic temporal lobe epilepsy induced by electrical stimulation of the amygdala in rat. Epilepsy Res. 38: 177–205. 10642046 10.1016/s0920-1211(99)00088-1

[64] Flannery, B.M. , D.A. Bruun , D.J. Rowland , *et al* 2016 Persistent neuroinflammation and cognitive impairment in a rat model of acute diisopropylfluorophosphate intoxication. J. Neuroinflammation 13: 267. 27733171 10.1186/s12974-016-0744-y PMC5062885

[65] Kruger, L. , S. Saporta & L.W. Swanson . 1995 Photographic Atlas of the Rat Brain: the Cell and Fiber Architecture Illustrated in Three Planes with Stereotaxic Coordinates. New York: Cambridge University Press 299 pp.

[66] Chemical Hazards Emergency Medical Management . CHEMM (US Department of Health and Human Services. Accessed June 1, 2020. https://chemm.nlm.nih.gov/. .

[67] Gustafsson, L.L. , W.F. Ebling , E. Osaki & D.R. Stanski . 1996 Quantitation of depth of thiopental anesthesia in the rat. Anesthesiology 84: 415–427. 8602674 10.1097/00000542-199602000-00021

[68] Lee, V.C. , J.C. Moscicki & C.A. DiFazio . 1998 Propofol sedation produces dose‐dependent suppression of lidocaine‐induced seizures in rats. Anesth. Analg. 86: 652–657. 9495432 10.1097/00000539-199803000-00040

[69] Trinka, E. , H. Cock , D. Hesdorffer , *et al* 2015 A definition and classification of status epilepticus—Report of the ILAE Task Force on Classification of Status Epilepticus. Epilepsia 56: 1515–1523. 26336950 10.1111/epi.13121

[70] Pouliot, W. , S.L. Bealer , B. Roach & F.E. Dudek . 2016 A rodent model of human organophosphate exposure producing status epilepticus and neuropathology. Neurotoxicology 56: 196–203. 27527991 10.1016/j.neuro.2016.08.002 PMC5048581

[71] Hobson, B.A. , D.J. Rowland , S. Supasai , *et al* 2018 A magnetic resonance imaging study of early brain injury in a rat model of acute DFP intoxication. Neurotoxicology 66: 170–178. 29183789 10.1016/j.neuro.2017.11.009 PMC5940565

[72] Kantanen, A.M. , R. Kälviäinen , I. Parviainen , *et al* 2017 Predictors of hospital and one‐year mortality in intensive care patients with refractory status epilepticus: a population‐based study. Crit. Care 21: 71. 28330483 10.1186/s13054-017-1661-x PMC5363025

[73] Rojas, A. , T. Ganesh , W. Wang , *et al* 2020 A rat model of organophosphate‐induced status epilepticus and the beneficial effects of EP2 receptor inhibition. Neurobiol. Dis. 133: 104399. 30818067 10.1016/j.nbd.2019.02.010 PMC6708729

[74] Reddy, D.S. & M.A. Rogawski . 2001 Enhanced anticonvulsant activity of neuroactive steroids in a rat model of catamenial epilepsy. Epilepsia 42: 337–344. 11442150 10.1046/j.1528-1157.2001.10200.x

[75] Frye, C.A. & L.E. Bayon . 1999 Prenatal stress reduces the effectiveness of the neurosteroid 3α,5α‐THP to block kainic‐acid‐induced seizures. Dev. Psychobiol. 34: 227–234. 10204098

[76] Hanada, T. , Y. Hashizume , N. Tokuhara , *et al* 2011 Perampanel: a novel, orally active, noncompetitive AMPA‐receptor antagonist that reduces seizure activity in rodent models of epilepsy. Epilepsia 52: 1331–1340. 21635236 10.1111/j.1528-1167.2011.03109.x

[77] Zolkowska, D. , A. Dhir , C. Banks , *et al* 2012 Perampanel, a potent AMPA receptor antagonist, protects against tetramethylenedisulfotetramine‐induced seizures. Epilepsy Curr. 12(Suppl. 1): 304. 10.1007/s00204-021-03053-9 PMC8241714 33914090

[78] Zolkowska, D. , C. Boosalis , D.A. Bruun , *et al* 2015 Perampanel, a potent noncompetitive AMPA receptor antagonist, enhances survival in tetramethylenedisulfotetramine‐induced status epilepticus. Abstract 2.242, American Epilepsy Society Annual Meeting. Accessed August 16, 2020. www.aesnet.org.

[79] Hanada, T. , K. Ido & T. Kosasa . 2014 Effect of perampanel, a novel AMPA antagonist, on benzodiazepine‐resistant status epilepticus in a lithium‐pilocarpine rat model. Pharmacol. Res. Perspect. 2: e00063. 25505607 10.1002/prp2.63 PMC4186423

[80] Kuruba, R. , X. Wu & D.S. Reddy . 2018 Benzodiazepine‐refractory status epilepticus, neuroinflammation, and interneuron neurodegeneration after acute organophosphate intoxication. Biochim. Biophys. Acta Mol. Basis Dis. 1864(9 Pt B): 2845–2858. 29802961 10.1016/j.bbadis.2018.05.016 PMC6066461

[81] Spampanato, J. , W. Pouliot , S.L. Bealer , *et al* 2019 Antiseizure and neuroprotective effects of delayed treatment with midazolam in a rodent model of organophosphate exposure. Epilepsia 60: 1387–1398. 31125451 10.1111/epi.16050 PMC6662604

[82] Bruun, D.A. , M. Guignet , D.J. Harvey & P.J. Lein . 2019 Pretreatment with pyridostigmine bromide has no effect on seizure behavior or 24 hour survival in the rat model of acute diisopropylfluorophosphate intoxication. Neurotoxicology 73: 81–84. 30853371 10.1016/j.neuro.2019.03.001 PMC6634995

[83] Capacio, B.R. , C.E. Byers , K.A. Merk , *et al* 2004 Pharmacokinetic studies of intramuscular midazolam in guinea pigs challenged with soman. Drug Chem. Toxicol. 27: 95–110. 15198070 10.1081/dct-120030727

[84] Amzica, F. 2009 Basic physiology of burst‐suppression. Epilepsia 50(Suppl. 12): 38–39. 19941521 10.1111/j.1528-1167.2009.02345.x

[85] Wang, M.D. , G. Wahlström , K.W. Gee & T. Bäckström . 1995 Potency of lipid and protein formulation of 5α‐pregnanolone at induction of anaesthesia and the corresponding regional brain distribution. Br. J. Anaesth. 74: 553–557. 7772431 10.1093/bja/74.5.553

[86] Zhu, D. , M.D. Wang , T. Bäckström & G. Wahlström . 2001 Evaluation and comparison of the pharmacokinetic and pharmacodynamic properties of allopregnanolone and pregnanolone at induction of anaesthesia in the male rat. Br. J. Anaesth. 86: 403–412. 11573532 10.1093/bja/86.3.403

[87] Phabphal, K. , S. Chisurajinda , T. Somboon , *et al* 2018 Does burst‐suppression achieve seizure control in refractory status epilepticus? BMC Neurol. 18: 46. 29679985 10.1186/s12883-018-1050-3 PMC5910581

[88] Paul, D. , L. Allakonda , A. Sahu , *et al* 2018 Pharmacokinetics and brain uptake study of novel AMPA receptor antagonist perampanel in SD rats using a validated UHPLC‐QTOF‐MS method. J. Pharm. Biomed. Anal. 149: 234–241. 29127904 10.1016/j.jpba.2017.11.008

[89] Martin, B.R. 1985 Biodisposition of [^3^H]diisopropylfluorophosphate in mice. Toxicol. Appl. Pharmacol. 77: 275–284. 3975900 10.1016/0041-008x(85)90327-8

[90] González, E.A. , A.C. Rindy , M.A. Guignet , *et al* 2020 The chemical convulsant diisopropylfluorophosphate (DFP) causes persistent neuropathology in adult male rats independent of seizure activity. Arch. Toxicol. 94: 2149–2162. 32303805 10.1007/s00204-020-02747-w PMC7305973

[91] Collombet, J.M. , C. Masqueliez , E. Four , *et al* 2006 Early reduction of NeuN antigenicity induced by soman poisoning in mice can be used to predict delayed neuronal degeneration in the hippocampus. Neurosci. Lett. 398: 337–342. 16472911 10.1016/j.neulet.2006.01.029

[92] Guignet, M. & P.J. Lein . 2019 Organophosphates In: Advances in Neurotoxicology: Role of Inflammation in Environmental Neurotoxicity. Vol. 3 Aschner M. & Costa L.G., Eds.: 35–79. Cambridge, MA: Academic Press.

[93] Nik, A.M. , B. Pressly , V. Singh , *et al* 2017 Rapid throughput analysis of GABA_A_ receptor subtype modulators and blockers using DiSBAC_1_(3) membrane potential red dye. Mol. Pharmacol. 92: 88–99. 28428226 10.1124/mol.117.108563 PMC5452057

[94] Cao, Z. , B.D. Hammock , M. McCoy , *et al* 2012 Tetramethylenedisulfotetramine alters Ca^2+^ dynamics in cultured hippocampal neurons: mitigation by NMDA receptor blockade and GABA_A_ receptor‐positive modulation. Toxicol. Sci. 130: 362–372. 22889812 10.1093/toxsci/kfs244 PMC3529644

[95] Bruun, D.A. , Z. Cao , B. Inceoglu , *et al* 2015 Combined treatment with diazepam and allopregnanolone reverses tetramethylenedisulfotetramine (TETS)‐induced calcium dysregulation in cultured neurons and protects TETS‐intoxicated mice against lethal seizures. Neuropharmacology 95: 332–342. 25882826 10.1016/j.neuropharm.2015.03.035 PMC4466064

[96] Chuang, S.H. & D.S. Reddy . 2020 Isobolographic analysis of antiseizure activity of the GABA Type A receptor‐modulating synthetic neurosteroids brexanolone and ganaxolone with tiagabine and midazolam. J. Pharmacol. Exp. Ther. 372: 285–298. 31843812 10.1124/jpet.119.261735 PMC7011113

